# Oceanographic anomalies coinciding with humpback whale super-group occurrences in the Southern Benguela

**DOI:** 10.1038/s41598-021-00253-2

**Published:** 2021-10-22

**Authors:** Subhra Prakash Dey, Marcello Vichi, Giles Fearon, Elisa Seyboth, Ken P. Findlay, Jan-Olaf Meynecke, Jasper de Bie, Serena Blyth Lee, Saumik Samanta, Jan‐Lukas Menzel Barraqueta, Alakendra N. Roychoudhury, Brendan Mackey

**Affiliations:** 1 Department of Oceanography, University of Cape Town, Rondebosch, 7701 South Africa; 2 Marine and Antarctic Research Centre for Innovation and Sustainability, University of Cape Town, Rondebosch, 7701 South Africa; 3 Centre for Sustainable Oceans, Faculty of Applied Sciences, Cape Peninsula University of Technology, Cape Town, South Africa; 4 Griffith Climate Change Response Program, Griffith University, Southport, Qld Australia; 5 Coastal and Marine Research Centre, Griffith University, Southport, Qld Australia; 6 Earth Sciences, Stellenbosch University, Cape Town, South Africa

**Keywords:** Ocean sciences, Animal migration

## Abstract

Seasonal feeding behaviour of humpback whales (*Megaptera novaeangliae*) has been observed in the coastal waters of the Southern Benguela where the species has been observed forming super-groups during the austral spring in recent years since 2011. Super-groups are unprecedented densely-packed aggregations of between 20 and 200 individuals in low-latitude waters and their occurrences indicate possible changes in feeding behaviour of the species. We accessed published data on super-groups occurrence in the study area in 2011, 2014 and 2015, and investigated oceanographic drivers that support prey availability in this region. We found that enhanced primary production is a necessary but not sufficient condition for super-groups to occur. Positive chlorophyll anomalies occurring one month prior to the super-group occurrences were identified, but only a concurrent significantly reduced water volume export from the region throughout October were conducive to the aggregations in the specific years. Hydrodynamic model results attributed the anomalous decreased volume export to the strength and orientation of the Goodhope Jet and associated eddy activity. The combination of random enhanced primary production typical of the region and emerging anomalous conditions of reduced water export in October since 2011 resulted in favourable food availability leading to the unique humpback whale aggregations. The novelty of this grouping behaviour is indicative of the lack of such oceanographic conditions in the past. Given the recency of the events, it is difficult to attribute this reduction in ocean transport to climatic regime shifts, and the origin should be likely investigated in the distant water mass interaction with the greater Agulhas system rather than in local intensifications of the upwelling conditions. A positive trend in the humpback whale population abundance points to the need to monitor the exposure of the species to the changing climate conditions.

## Introduction

**Figure 1:**
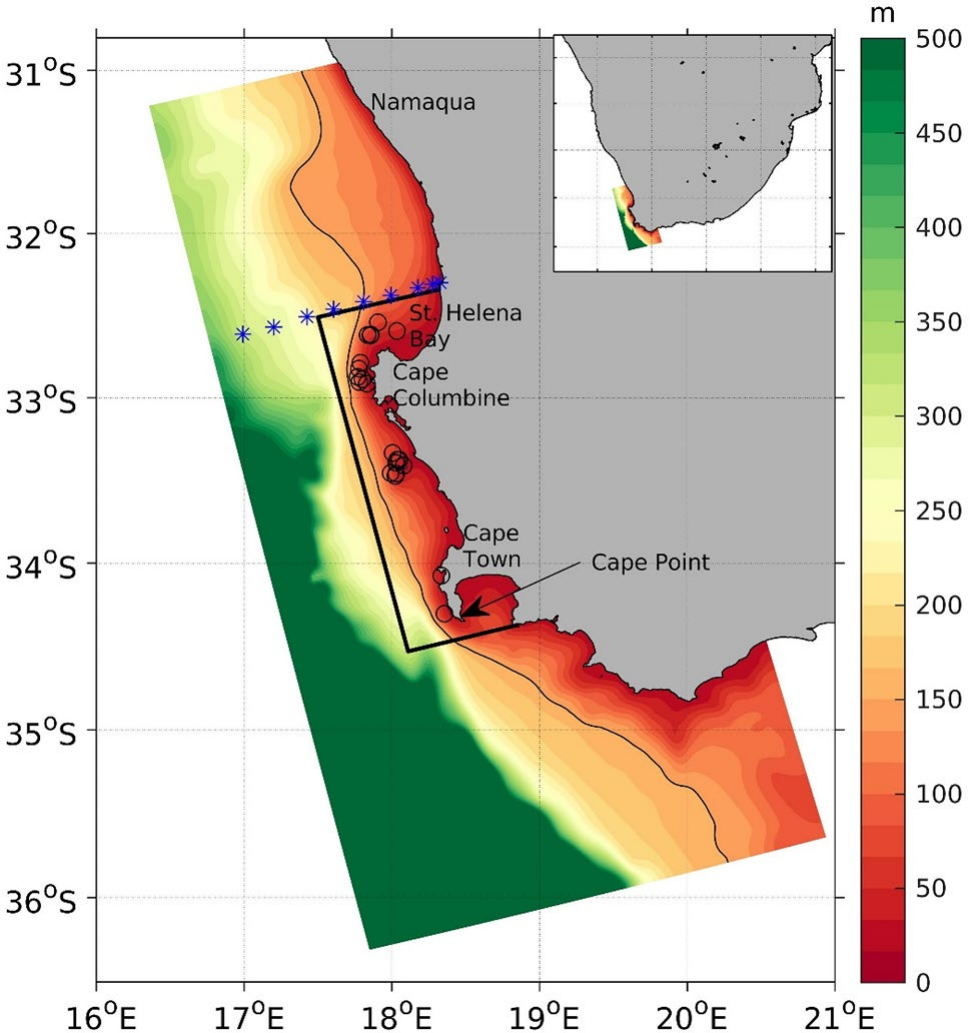
The region where humpback whale super-groups have been observed within the Southern Benguela Upwelling System (SBUS). The color shading represents the bathymetry of the ocean model domain. The black contour indicates the 150 m isobath. The open circles represent the locations of super-groups observed in 2011, 2014, and 2015 (data from Findlay et al.^9^). The ocean area enclosed by the rectangle covers all the super-group locations and is termed the focus area. The blue asterisks show the St Helena Bay Monitoring Line with the nine fixed stations from the coast to offshore. The inset picture shows the southern part of the African continent and the location of the model grid (color shade in inset image). This figure is plotted using MATLAB 2020b (https://matlab.mathworks.com/) with M_Map (a mapping package available at https://www.eoas.ubc.ca/~rich/map.html).

Humpback whales (*Megaptera novaeangliae*) in the Southern Hemisphere are well-known for their annual migrations between the summer high-latitude Southern Ocean feeding grounds and the winter mating and calving grounds in low-latitude, tropical and subtropical coastal waters^1–3^. The distribution of humpback whales at low-latitude mating and calving grounds during the Austral winter is highly correlated with sea surface temperature (SST) ranging between 21.1–28.3 °C in relatively shallow waters^3^. Food availability is the main determinant of the distribution of the animals during the Austral summer^4,5^. Feeding behaviour outside the Southern Ocean is less common, but has been documented^6–8^. Since 2011, densely packed feeding individuals (ranging 20–200), termed ‘super-groups’^9^, have been observed in the coastal region of the Southern Benguela between St Helena Bay and Cape Point (Fig. 1) from late October to early November in apparently random years (2011, 2014, and 2015, according to the published results). This feeding strategy at such low latitudes (ranging 32.5°S–34.5°S) is unprecedented in this region, based on the current observational knowledge and historical documents from the whaling era^9^. Considering the global observational records, it was unprecedented until recently, when super-groups have also been reported in Australia^10^. This raises the question about the possible drivers of such events and the likelihood that they would become permanent. Findlay et al.^9^ hypothesized that individuals partaking in super-groups belong to the Breeding Stock C along with some non-breeding young individuals migrating from Antarctic waters. The aggregations may be caused by either the recovering population of humpback whales placing pressure on prey availability elsewhere, alterations in prey availability leading to a novel feeding behaviour, or a restoration of a previously unobserved feeding strategy as the population recovers after whaling pressure. A further hypothesis is linked to the emergence of a regime shift of oceanographic conditions in the region dominated by upwelling. The super-groups may form due to the above-mentioned factors or a combination of these.

The Southern Benguela Upwelling System (SBUS) is a highly productive region, which includes three upwelling cells: Namaqua, Cape Columbine, and Cape Peninsula^11^ (Fig. 1). The upwelling in these cells is highest in austral spring/summer, attributed to the south-eastward movement of the South Atlantic Anticyclone^12^. The early spring period partly overlaps with the southward migration of humpback whales at this time of year, although both the lack of observed calves and the incidence of cold water skin diatoms^13^ suggest super-group whales have not recently migrated from more northerly African west coast breeding grounds^9^.

The distribution of potential prey of humpback whales in the Southern Benguela is theoretically dependent on a variety of conditions: the upwelling intensity, the associated resulting primary and secondary productions, and the advection/dispersion by the Benguela current and associated eddies. Upwelled water supplies nutrients to the euphotic zone which supports phytoplankton growth. The blooms nourish zooplankton communities, including euphausiids^14^, one of the main known food sources for humpback whales in the region^15^. Zooplankton biomass supports small pelagic fish abundances (e.g., South African sardine, *Sardinops sagax*, and anchovy, *Engraulis encrasicolus*) that feed higher trophic-levels^16^. The phenology of zooplankton abundance is known to vary considerably in relation to water temperature and primary production^17,18^. In the SBUS, such phenology is characterised by a clear seasonal cycle, with a winter minimum and a spring/summer maximum when phytoplankton is most abundant^19,20^, although dedicated studies on the time-lag between primary and secondary plankton production are not available and the modelling focused mostly on annual trends^21^. A tendency toward increasing upwelling in the SBUS was noted by several studies^22–24^ in recent years. Conversely, Lamont et al.^25^ reported a decreasing trend in chlorophyll in the southern Benguela shelf region by season over the last 20 years.

The food availability for the higher trophic level in the SBUS is also dependent on the retention of primary and secondary production inside the region, which is attributed to the orientation and strength of the current system^26^. The Benguela current^27^ flows equatorward as the southern limb of the South Atlantic subtropical gyre which receives south Indian Ocean waters from the Agulhas current. The mean state of the Benguela current has two main patterns: a topographically-controlled inshore stream which follows the shelf-edge, and the offshore stream which is driven by nonlinear interactions with the Agulhas rings^28^. The Southern Benguela is characterized by a wide shelf and moderate to high eddy activity^29^. The high eddy activity in other eastern boundary upwelling systems such as the California Current is known to suppress primary production^30^. In the SBUS, the Agulhas rings result in periodical offshore advection of phytoplankton, fish eggs and larvae^31,32^.

Though the SBUS has always been a highly productive region^20^, humpback whale super-groups were not observed before 2011. Baleen whales including the species humpback whale were monitored through extensive shore-based studies carried out in the Cape Columbine region (Fig. 1) since 1995^6,7,33,34^. This type of monitoring is capable of observing a large group of individuals aggregating in the same way the super-groups did since 2011. Reports of humpback whales feeding in the region (which is somewhat unique compared to other migration corridors) have been recorded by a number of authors. For example, Findlay and Best^8^ reported on a juvenile entanglement mortality that had surprisingly fed on stomatopod prey (a predominantly burrowing species, which may swarm feed at times). Best et al.^7^ reported on a number of observations of feeding in the SBUS as did Barendse et al.^6^. Prey selection in these studies centred on krill *Euphausia lucens*, but there are also evidences of the amphipod *Themisto gaudichaudi*^34^, as it was sampled around feeding groups of the species in the west coast of South Africa^35^, as well as small fishes, as observed in the stomach of an individual of the species taken in Saldanha Bay during the whaling era^36^. The formation of super-groups in recent years suggest that there might be a change in oceanographic or ecological characteristics which provide the conditions for this novel feeding strategy in the SBUS^9^.

The research presented here is part of an interdisciplinary approach to understand the impacts of climate change on the recovering population of humpback whales^37^. This paper focuses on the possible environmental features connected to the super-group formation in the Southern Benguela. We propose that recent episodes of weakening of the highly seasonal and inter-annual nearshore stream of the Benguela current and associated eddy activities have contributed to the retention of higher phytoplankton biomass concentrated in a narrow coastal band during austral spring, the period routinely visited by humpback whales. Aggregations of the species may be further enhanced by the concurrent recovery of the population as a result of protection from severe whaling pressure last century. We support our findings by means of a regional model and an array of Earth observations and discuss the outcome in the context of possible climatic shifts in the highly variable SBUS.

## Results and discussions

Humpback whale super-groups^9^ were reported during late October and early November in 2011, 2014, and 2015. They occurred in a very localized coastal area within the 150 m isobath (Fig. 1), extending between St Helena Bay in the north and Cape Point. This region covering roughly 10,000 square km of coastal ocean area, is termed the focus area hereafter. The super-groups were found inside the focus area within a time window between 28th October to 14th November.

### Oceanographic drivers of favourable super-group conditions

Chlorophyll concentration is a proxy for phytoplankton biomass and has a potential to be an environmental driver impacting the baleen whale behaviour. The species humpback whale is most documented to show opportunistic feeding while migrating^38^ and phytoplankton blooms are thought to set the suitable foraging ground generating secondary production^39^. For instance, Trudelle et al.^40^ reported that the behavioral response of baleen whales in the coastal water near Madagascar were predicted best when chlorophyll was included in statistical models. In addition, the timing of the migratory baleen whales at the Azores is highly correlated with the North Atlantic spring bloom^39^.

**Figure 2:**
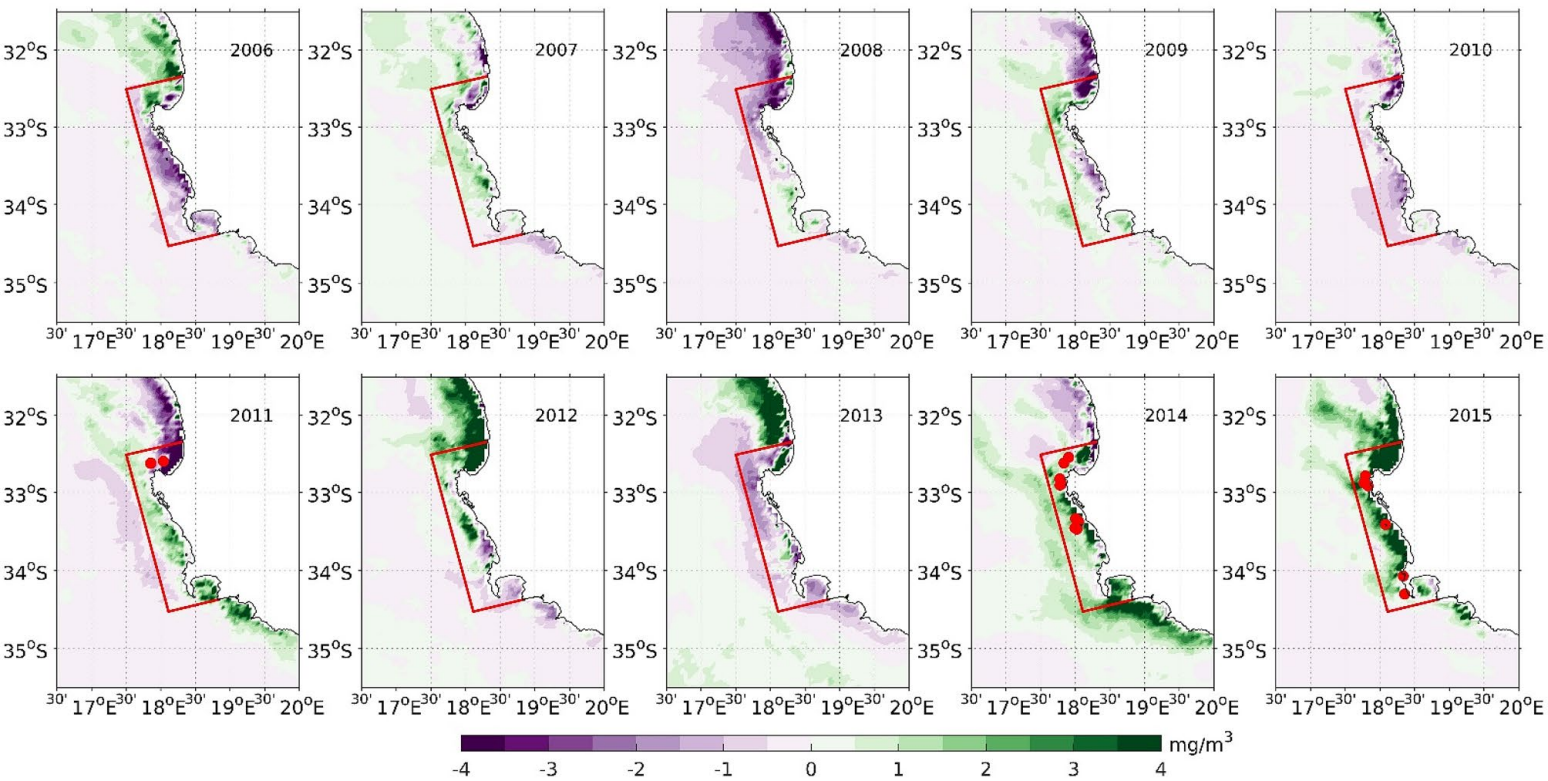
Chlorophyll-a monthly anomaly (in *mg/m*^*3*^) in October in years 2006 – 2015. The red dots represent the locations of the super-groups in 2011, 2014 and 2015. The area enclosed by the red lines represents the focus area. The plots are generated using MATLAB 2020b (https://matlab.mathworks.com/) with M_Map (a mapping package available at https://www.eoas.ubc.ca/~rich/map.html).

Remotely sensed chlorophyll concentration data (see “Methods”) allows us to analyse the interval of time around the years of super-groups occurrence. Phytoplankton size distribution is also available from space^25^, but since it was not specifically addressed in relation to super-groups, the focus was on the bulk chlorophyll. Figure 2 shows a comparison between the chlorophyll anomalies for October 2006–2015 with respect to an 18-year climatology (see “Methods”). A positive chlorophyll anomaly represents chlorophyll concentration higher than the climatological mean and the negative anomalies represent the reverse. Positive chlorophyll concentration anomalies are established which extend throughout the focus area in October 2011, 2014, and 2015 (Fig. 2). Some evidences of positive chlorophyll anomalies were also present in 2007, 2009 and 2012, in which there were no super-group sightings in the focus area. The September chlorophyll anomaly was slightly positive in 2011, negative in 2014, and slightly negative in 2015 inside the focus area (supplementary Fig. S1). The data therefore suggests that high chlorophyll in October represents favourable conditions for possible super-group formation. The wind stress in the Southern Benguela is predominantly south-easterly or southerly during October, contributing to coastal upwelling in the region. The analysis of wind stress obtained from the Advanced Scatterometer (ASCAT, see “Methods”) shows weak wind stress at the Southern Benguela region during October in 2010, 2011, and 2015 compared to other years, suggesting that these years were characterized by weak Ekman upwelling (Supplementary Fig. S2). Hence the wind stress conditions in the super-group years did not result in enhanced Ekman upwelling along the coastal SBUS and therefore do not explain the enhanced chlorophyll concentration in October. This suggests that other local ocean processes may be linked with the anomalous October phytoplankton blooms in the mentioned years.

**Figure 3:**
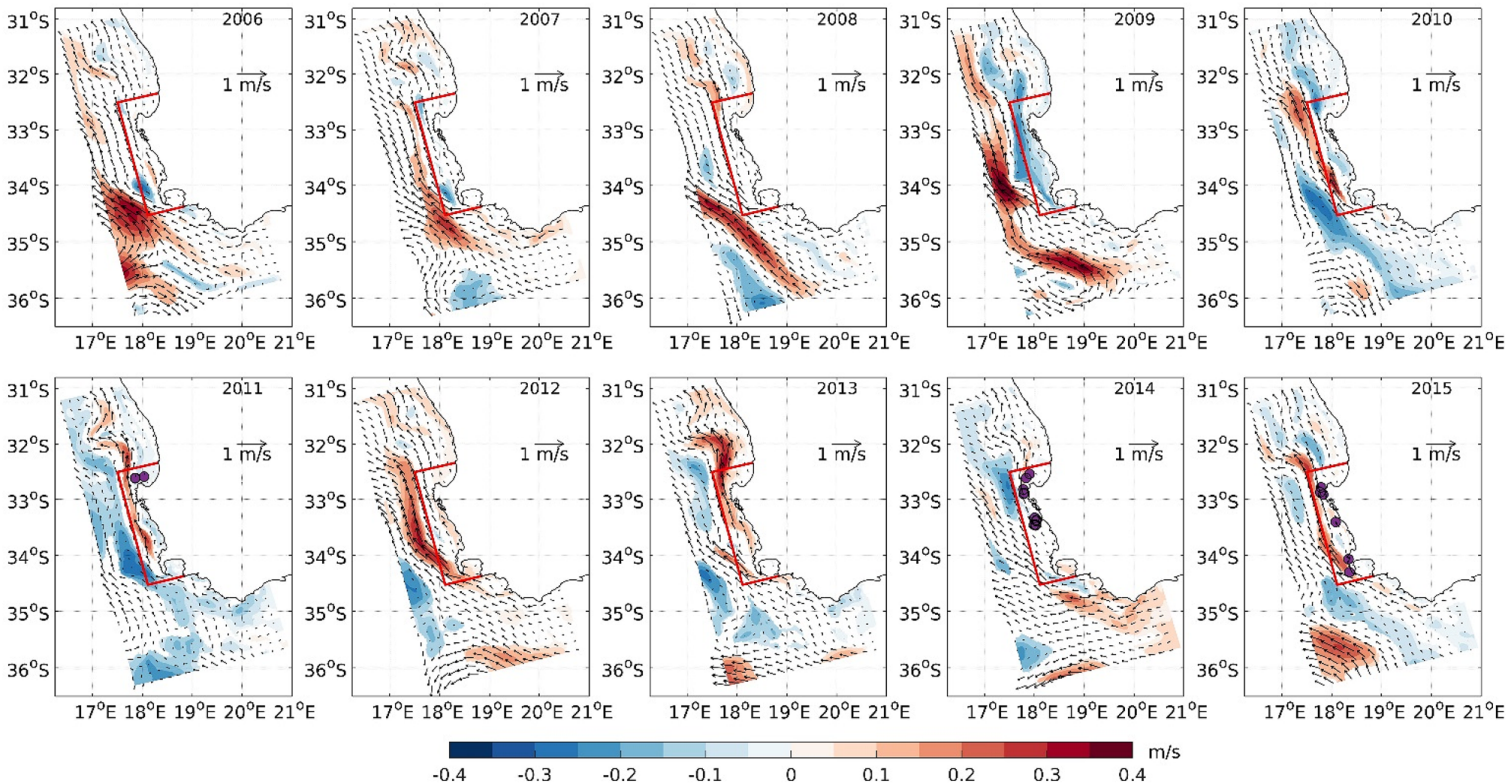
Mean October surface current in 2006–2015. The color shading represents the monthly anomaly (significant above 95% confidence level, in *m/s*) of current speed with respect to the 2006–2015 climatology and the overlying vectors represent the monthly mean current for that year. The dots represent the locations of the super-groups in respective years. The focus area is enclosed by the red lines. The plots are generated using MATLAB 2020b (https://matlab.mathworks.com/) with M_Map, a mapping package available at https://www.eoas.ubc.ca/~rich/map.html.

To understand the possible physical drivers of primary production change and the connection with super-group occurrence, we turn our attention to the results of the regional ocean model: Coastal and Regional Ocean COmmunity model (CROCO) that was run over the period 2006–2015 (see “Methods”). The primary and secondary productions are highly dependent on ocean currents. Figure 3 shows the mean October surface current (vector) and speed anomaly (color) with respect to the climatology (Supplementary Fig. S3) for different years. The speed anomalies are significant above 95% confidence levels (see “Methods”). The system of shelf-edge jet currents is a subset of the southern limb of the Benguela current. The prominent velocity feature observed between 32–35°S in Figure 3 is called the Goodhope Jet^41^. This feature encompasses both the Cape Peninsula (to the South) and Cape Columbine Jets (to the North), which are both characterised by large temporal variability. The Goodhope Jet was narrower, weaker and shifted towards the coastline in 2011 and 2015. In 2014 while the current remained weaker than the climatology, its width increased compared to 2011/2015. Generally, the region near the Agulhas Bank is less productive, having lower chlorophyll concentration compared to the focus region because of the positions of the upwelling cells^11,42^. Hence the chlorophyll concentration in the focus region is more likely to be affected by the local processes rather than the southern boundary inflow. The orientation of the alongshore, northward-flowing Goodhope Jet suggests that the outward flowing water from the focus region through its northern and western boundaries contribute most to the primary and secondary production accumulation. For instance, in the years of high chlorophyll anomaly 2007 and 2012, the Goodhope Jet was broader, stronger and shifted offshore, passing through the longer western boundary of the focus region (Fig. 3). In 2009, another year of anomalous high October chlorophyll, the Goodhope Jet was far offshore and no part of it flowed through the focus region. The current in 2013 was similar to 2015 but the chlorophyll anomaly inside the focus region was negative.

**Figure 4:**
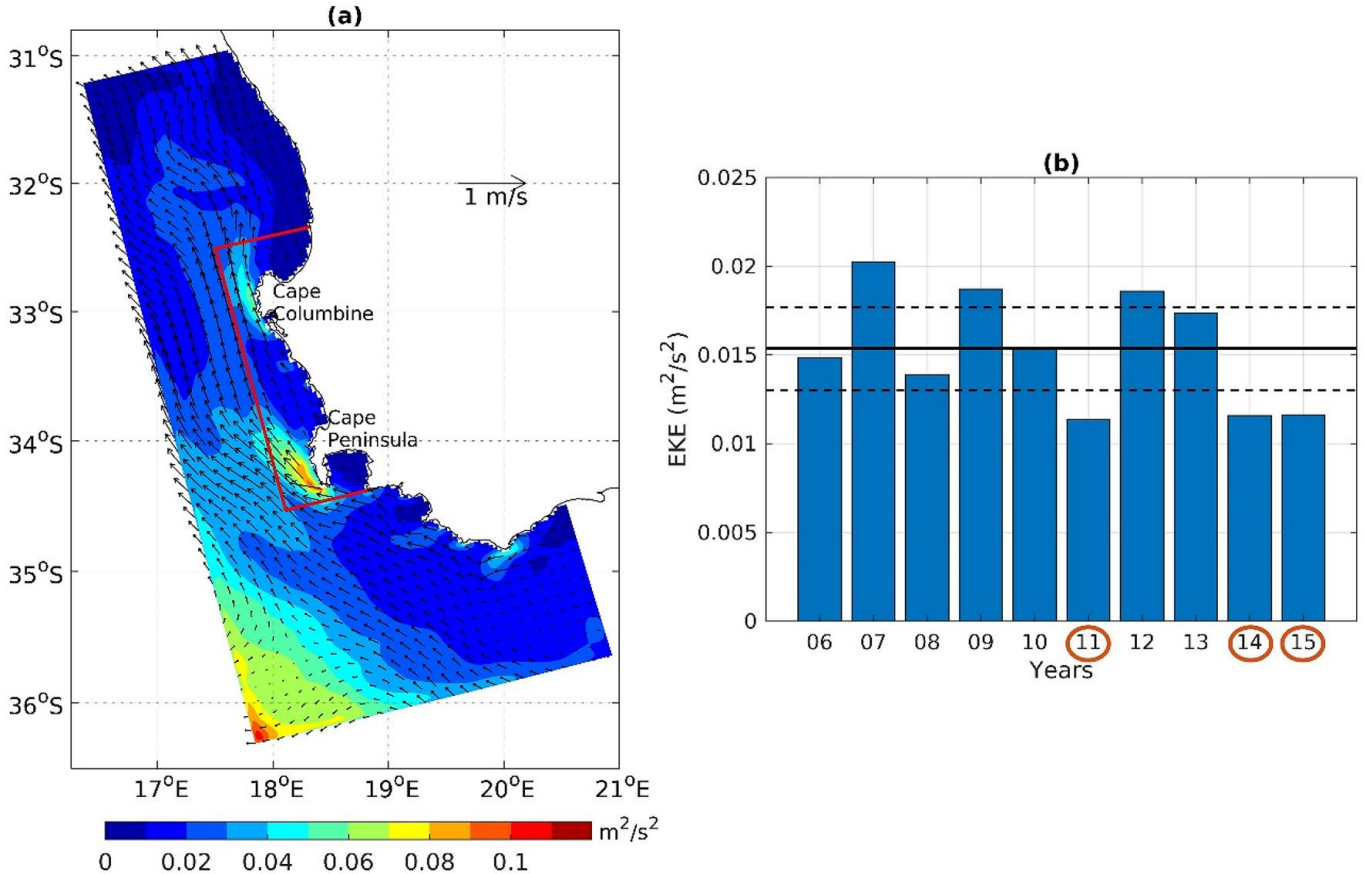
(**a**) Mean surface Eddy Kinetic Energy (EKE) (shading, in *m*^*2*^*/s*^*2*^) and current in October estimated from the CROCO simulation. Note the high EKE patches in the coastal regions near Cape Peninsula and Cape Columbine. (**b**) Average October EKE in the focus region in different years. The continuous line shows the mean of all the years and the dotted lines represent the 95% confidence interval. The encircled years in x-axis represent the years of super-groups. The plots are generated using MATLAB 2020b (https://matlab.mathworks.com/). A mapping package M_Map (https://www.eoas.ubc.ca/~rich/map.html) is used for panel (**a**).

Visual interpretation of interannual variation in the October chlorophyll and current patterns suggests that high chlorophyll and weak Goodhope Jet are associated with reduced offshore export/flushing of phytoplankton biomass, which may set up favourable conditions for super-group occurrence. However, it is difficult to interpret the flushing mechanism only by the monthly mean surface current. Generally, the SBUS is dominated by moderate to high eddy activity compared to the rest of the Benguela upwelling system^31,32^. High eddy activity has been demonstrated to flush upwelled nutrients and primary production offshore, away from upwelling systems^30^. Figure 4a shows the mean eddy kinetic energy (EKE) and surface currents in October estimated from the CROCO simulation (see “Methods”). Two patches of high EKE are noted inside the focus region, one near Cape Columbine and the other near Cape Peninsula, which correspond to the two components of the Goodhope Jet^41^. The October EKE is significantly lower than average in all the super-group years: 2011, 2014 and 2015 (Fig. 4b), leading to decreased offshore transport from the focus region. Less than average EKE was also noted in 2008 though it was not significantly anomalous like the super-group years, while the chlorophyll anomaly was negative in this year (Fig. 2). The eddy activity was the highest in 2007 and second highest in 2009. Though the Goodhope Jet does not cross the boundaries of the focus region in 2009, the offshore eddy transport is high, which would then contribute to the quick dispersion of the biomass. The quantitative link between the biomass variation due to flushing and super-group occurrence will become more evident from a detailed analysis of outward water volume transport and corresponding chlorophyll concentration, presented in the next section.

### Indices for super-group occurrences

**Figure 5:**
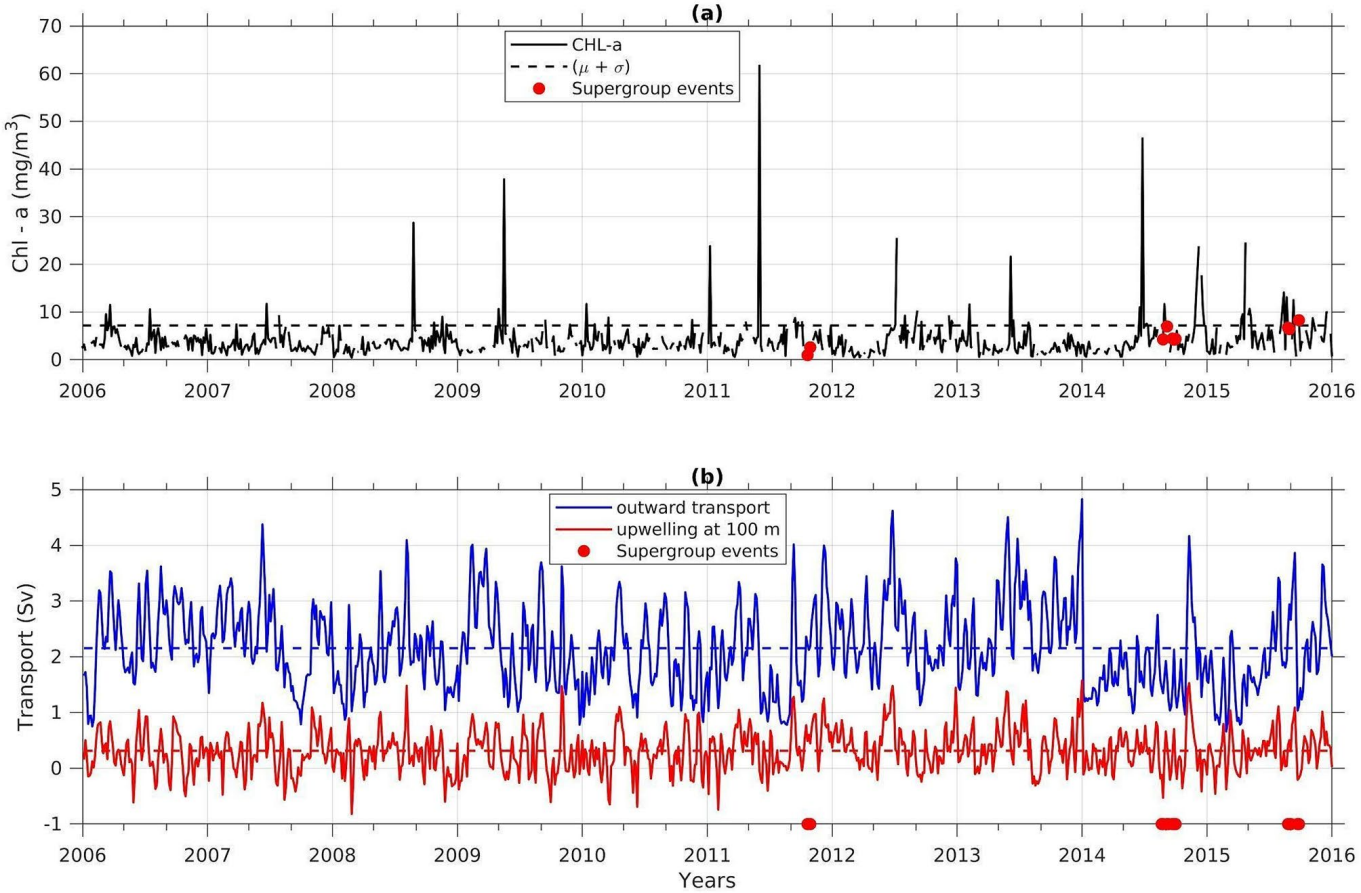
(**a**) Satellite-derived chlorophyll-a (mg/m^3^) time series averaged over the focus area. The dashed line represents the one-standard-deviation level above the mean chlorophyll. (**b**) Simulated total outward transport (blue line, in Sv) from the focus area and total upwelled water at 100 m depth (red line, in Sv). The dashed blue and red lines represent the mean outward transport and mean upwelling, respectively. Only three months—September, October, and November are plotted in each year in both the plots. The red dots in both panels represent the occurrence of the super-group events.

Figure 5a shows the time series of satellite-derived chlorophyll-a averaged over the focus area for the period 2006–2015. Chlorophyll-a data is used to detect the phytoplankton blooms. Since the super-groups were sighted in the period 28 October–14 November, only the September–November months of each year are presented. This plot identifies the occurrence and persistence of the phytoplankton blooms that lead to the October anomalies highlighted in Fig. 2. A large phytoplankton bloom occurred around one month prior to each super-group occurrence. These blooms are significantly large, exceeding the one-standard-deviation line, which is more than 99% confidence level above the mean. Though the bloom one month prior to the 2015 sighting was smaller compared to other years of super-group events, two chlorophyll peaks exceeding one standard deviation line were recorded during the end of September and mid-October, respectively. We observe a lag between the peaks and the super-group events that is hypothesized to be due to ecological interactions between the different trophic levels. This ecological relationship is likely to be different in different regions, and the specific lag in the focus area cannot be quantified due to limited historical data on zooplankton at the sub-seasonal scale. The existing model applications that include higher trophic levels are focused on the annual recruitment^21^, and hence ignore the seasonal details. Studies on humpback whales in other regions show that a minimum of one month lag is required to grow the zooplankton after the primary production peak. For instance, co-occurrence of chlorophyll, zooplankton and krill biomass and preference for high chlorophyll peaks have been reported in the literature^5,43^, with indications that humpback whales target krill early in the season in the California Current upwelling system^43^. Longer-term data from Hayward and Venrick^44^ reported 1–4 months delay between chlorophyll-a and macrozooplankton peaks in the California upwelling system. Another study on blue whales in that same region based on one year of observations indicate that peak euphausiid densities and whale predation lag primary production by 3 to 4 months^45^. In the Benguela, the lag can depend on a number of factors affecting phytoplankton speciation and humpback whale prey. Small size flagellates dominate in-shore of the 130 m isobath^46^, where whales aggregate. These flagellates also breakdown more readily, thus becoming available for zooplankton grazing at shorter time scales. There is evidence of daytime swarms of euphausiid in Southern Benguela between August and November^47^. In October, sardines (another possible source of food for humpback whales^48^) dominate in the region^49^. Based on the existing knowledge, one month length is considered a minimum time lag that would set the ecological conditions for humpback whale aggregations in the region.

Continuing with the analysis of the oceanographic drivers, the blue curve in Fig. 5b shows the simulated total outward volume transport from the focus area through all the three open boundaries via the top 100 m of the water column (see “Methods”). This quantity includes both the advective and eddy-related transport. The outward transport remained lower than average (Fig. 5b) after the bloom (Fig. 5a) up until the super-group events in all three years. Outward transport of water at the northern boundary of the focus area is dominated by the Goodhope Jet export intensity, while at the western boundary it is dependent on its westward shift, broadening and eddy transport (Fig. 3). Overall, the outbound water export is maximum through the western boundary because of its larger length, while it is minimum at the southern boundary due to the northward-flowing Goodhope Jet (Supplementary Fig. S4). The red curve in Fig. 5b shows the simulated upwelled/downwelled water in Sv at 100 m depth, averaged over the analysis region. It is noteworthy that the chlorophyll peaks do not always coincide with the upwelling. This may happen because we considered only a sub-region of the Benguela upwelling system. Moreover, the mean outward transport is ~ 7 times larger than the mean rate of supply of the upwelled water, resulting in rapid flushing of the upwelled nutrients, and low retention rates in the region. This further explains the lack of simultaneous peaks in upwelling and chlorophyll inside the focus area.

**Figure 6:**
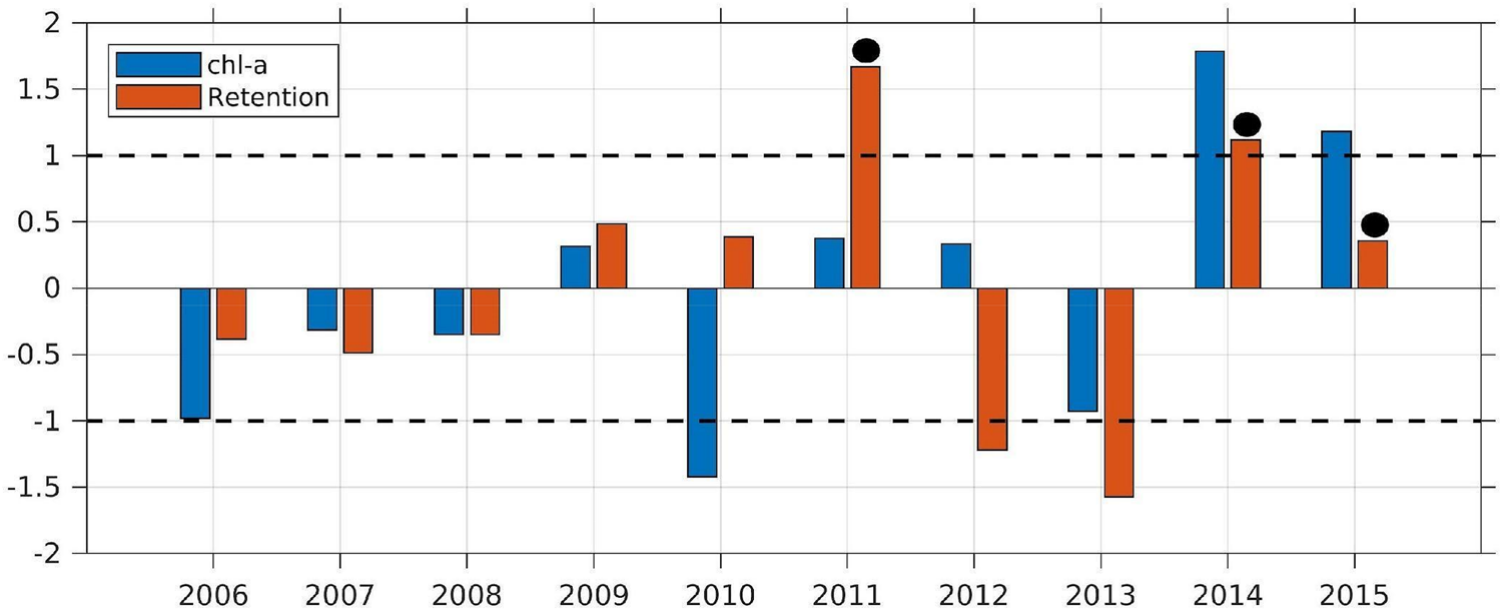
Normalized chlorophyll-a (chl-a; blue bars) and retention (red bars) averaged in the focus area in October. The dashed lines represent the 1-standard-deviation levels. The black dots over the red bars represent the years of humpback whale super-group events.

Figure 6 presents the interannual variation of the normalized chlorophyll and retention indices for October that are proposed to explain the occurrence of the super-groups. The computation of these indices is explained in the "Methods" section. The chlorophyll index is positive in 2009, 2011, 2012, 2014, and 2015. The retention was significantly negative in 2012, implying that the produced biomass flushed out from the region. The analysis of both the indices suggests the following criteria for the occurrence of super-group events:

- Both the indices have to be positive; and
- At least one of the indices should cross the one standard deviation limit. This means that either the chlorophyll or the retention inside the focus area must be significantly higher than the average.

Both the chlorophyll and retention indices are below the one standard deviation limit in 2009 resulting in no aggregation. This analysis indicates that any phytoplankton bloom or enhanced primary production in October inside the focus area would eventually trigger sustained prey availability for humpback whales’ aggregation only if the outward transport from the region is significantly low. The retention index presented here is different from the conventional topographic retention that occurs in the St Helena Bay area^50^. The enhanced retention is due to the intermittency of the continuous outward transport from the focus region, which is a characteristic of upwelling regions^30^ but that until now it was not associated to behavioural responses of higher trophic levels.

Both the retention and chlorophyll indices show higher amplitude from 2011 and produce frequent favourable conditions for super-groups during the austral spring months when humpback whales are migrating from breeding to feeding grounds (Fig. 6). This results in opportunistic feeding behaviour for the super-groups to adapt to prey scarcity, possibly arising from the increasing population identified by Branch^51^ and Findlay et al.^52^.

### Emerging climatic changes and super-groups

At this stage, it is difficult to confirm if the presented oceanographic anomalies preconditioning the super-groups occurrence are emerging climatic features because the events only occurred since 2011. There are concerns in detecting climatic signals using relatively short time series from Earth observations^53^. Any detection of a tipping point involving higher trophic levels would require at least a decade of data before and after the inception point.

The super-group data utilized here are the only published results derived from dedicated whale-observation cruises, which were organized in conjunction with incidental observations^9^. The lack of reports of super-groups prior to 2011 requires justification in terms of the possible lack of the observation effort. Humpback whale super-groups are highly visual to ocean users and often occur within visual range from the coast. Observations of supergroups have been reported to citizen science datasets or to the authors on numerous occasions since 2011. No reports were received prior to 2011 and the authors are confident that there has been no marked change in citizen science observer effort that would increase the reports of supergroups after 2011. However, the possibility that super-groups occurred in distant areas from the reported incidental sightings and our focus region cannot be ruled out. After 2015, several super-group occurrences were reported in citizen science databases. These datasets have not been included in this study due to their anecdotal characteristics, which should undergo careful scrutiny before scientific usage. We also acknowledge the underlying link between the increased occurrence of this novel feeding behaviour and the population increase, but the complex and partially unresolved nature of the humpback whale population structure found in the region represents an open question that needs to be assessed. For instance, the migratory links between the humpback whales observed off west South Africa and tropical West Africa remain unresolved^6,54,55^, and it is not clear whether there may also be individuals from other Breeding Stocks joining the aggregations.

Nevertheless, given the oceanographic evidence, there is a possibility that super-groups would become a persistent feature, which draws attention to the role of climatic signals driving the observed phenomenon. The region has experienced regime shifts in the past; it went through a major shift from sardines to anchovies in the ‘60 s, which was most likely caused by overexploitation^56^, while other ecosystem changes occurred in the mid ‘90 s, which were hypothesised to be the result of environmental changes^49,57,58^.

Climate change may impact the Benguela upwelling system via several mechanisms. Bakun^59^ and Bakun et al.^16^ hypothesized the climate change-driven intensification of upwelling-favourable alongshore winds in eastern boundary upwelling systems. Wind intensification has been detected across most latitudes for the Benguela system, consistent with the warming pattern associated with climate change^60,61^. The alongshore wind intensification resulted in an increasing trend in upwelling indices in the Benguela, which does not always lead to enhanced primary production^23^, and can have less predictable sub-seasonal effects because the ecosystem impacts are usually assessed through models run at the annual scale^21^. Variability at the larger scale may be masked by contrasting local factors: moderate upwelling would support phytoplankton growth via upwelled nutrients; strong upwelling winds may lead to deep wind driven mixing, enhanced turbulence and increased light limitation, which may decrease primary production; and finally, as shown in this paper, oceanographic retention may affect the residence time of the produced biomass, further impacting on secondary production. In addition, it is hypothesised that climate change would intensify the land-sea breeze^16^, which can further enhance wind-driven mixing, particularly near the critical latitude of 30° N/S for diurnal-inertial resonance^62^. In the California Current system, modelling studies gave indications of complex non-linear responses of the simulated planktonic ecosystems to a future warming climate, given the range of different scales involved^63^. Anthropogenically-driven upper ocean warming has been shown to enhance stratification and suppress primary production in other upwelling regions such as the northern Indian Ocean^64^. These complex responses may also have unpredictable implications on the phenology and the time-lag between primary and secondary production, which can further modulate super-group events. Studies on climate change response have been undertaken in the SBUS at coarser and aggregated resolutions, with an emphasis on fisheries^65,66^. The results from these recent works indicate that stratification enhancement may counteract the intensified wind-driven mixing, with contrasting responses of the higher trophic levels. Thus, though the anticipated larger scale climatic signal is conducive to potential growth enhancement, there are several local factors that modulate it. In our analysis, we indeed observed that the upwelling peaks do not always coincide with chlorophyll-a peaks in Fig. 5.

We hypothesize that humpback whales are detecting a signal that is likely to be a combination of local and remote effects, and that it is unlikely to be captured by the coarser resolution of climate models (currently 25 km). Our model resolution (3 km, see “Methods”) is sufficient to simulate mesoscale eddies and indicates no need to further introduce smaller sub-mesoscale features to produce the interannual variations in turbulent eddy kinetic energy that support retention. To reinforce this conclusion, a portion of the simulated period until 2012 was repeated with a higher resolution atmospheric forcing known to produce more realistic wind conditions^62^, which resulted in equivalent mean ocean state and interannual variability (see “Methods”).

To further understand the impacts of climate change in this region and the link with higher trophic levels there is a need for sustained long-term observations, as for instance used in Rykaczewski and Checkley^67^, and concurrent modelling tools as employed in this work, but also a greater focus on transitional seasons, such as the early austral spring period from September to October, which is less studied in terms of long-term variability. The emergence of the super-groups and our analysis reveal the role of the Goodhope Jet and its potential biomass retention during this period. Modelling studies link the variability of the Jet to the interplay between the Agulhas and Benguela currents^68^. The Agulhas current is a potential climate trigger^69^, which has shown changes in its features over the past 30 years^70^. Whether the oceanographic feature we identified as a possible trigger for super-groups is driven by changes in the greater Agulhas system is a novel question that would require a dedicated research effort in the future.

## Conclusions

Combined satellite observations and a physical ocean model were used to understand environmental drivers of the unprecedented humpback whale aggregations in the Southern Benguela off South Africa. The most prominent necessary condition for the occurrence of super-groups that we can identify is the occurrence of an anomalous phytoplankton bloom during the month of October, within one month prior to super-group events. However, this condition alone is not sufficient: reduced outward oceanographic volume transport during the month of October is also required. This is attributed mainly to the orientation of the Goodhope Jet and the associated eddy activity. Both the retention and chlorophyll concentration are found to be significantly higher in October in 2011, 2014 and 2015 compared with other years. We hypothesize that the combination of phytoplankton bloom and reduction in water volume export from the area in October leads to an increase in secondary production and high concentrations of the humpback whale prey.

Though the detailed atmospheric and oceanographic settings explaining the origin of these anomalies remain unknown, this study provides evidence of causal events that would allow researchers and the whale watching industry to prepare for super-group occurrences at least one month in advance, through the evaluation of chlorophyll and retention indices. Further, this study proposes that the outlined oceanographic preconditioning during austral spring from 2011 favoured super-group formation, which may indicate an emergent climatic pattern in the Southern Benguela. Since the phenomenon is relatively new, it needs to be confirmed with longer time series, dedicated super-group survey data and possibly an analysis of climate model outputs that focuses on the Benguela-Agulhas system during the early austral spring period.

## Methods

### Remote sensing data and chlorophyll index

The chlorophyll data are obtained from the Ocean Colour Climate Change Initiative (OC-CCI) dataset, Version 4.0, European Space Agency (ESA, http://www.esa-oceancolour-cci.org/). This dataset was created by band-shifting, and bias correcting the MERIS, MODIS, and VIIRS data to match SeaWiFS data and then merging all of them. This is a daily product with 4 km of spatial resolution. The monthly chlorophyll anomalies analyzed in this study were estimated by removing the monthly climatology for the period 2001 to 2018 from the monthly mean. The chlorophyll index (*CI*) shown in Fig. 6 is computed by normalizing the chlorophyll concentration in the focus region using the following equation.

1 $$CI= \frac{chl- \overline{chl}}{{\sigma }_{chl}}$$

where *chl* is the chlorophyll concentration of October averaged over the focus region highlighted in Fig. 1. $\overline{chl }$ and ${\sigma }_{chl}$ denote the mean and standard deviation, respectively, of average October chlorophyll in all the years studied. It is necessary to check the data distribution before calculating the index. A two-sided goodness-of-fit test (Lilliefors Test) shows that the chlorophyll concentration at the focus area is normally distributed at 1% significance level and has a skewness of -0.03 (Lilliefors^71^). This suggests that the mean October chlorophyll is not biased towards the lower values and we may estimate the normalization index.

The monthly wind stress data used in this analysis are estimated from the daily averaged gridded surface wind fields obtained from Advanced Scatterometer (ASCAT, https://manati.star.nesdis.noaa.gov/products/ASCAT.php). Data are available for the period from April 2007 to present with a spatial resolution of 0.25°.

The super-group data (group size, time, and location) were obtained from Findlay et al.^9^. These data were collected during dedicated research cruises in 2011–2015.

### Model description

The Coastal and Regional Ocean COmmunity model (CROCO, http://www.croco-ocean.org/), an ocean modeling system built upon ROMS-Agrif^72,73^, is used in this study to model the Southern Benguela ocean state. The model simulates the full ocean hydrodynamics, from the wind-driven circulation to the thermal and salinity components that contribute to water density and the subsequent motion of different water masses (the baroclinic flow). CROCO is a three-dimensional, free-surface, terrain-following ocean model with split-explicit time stepping. It solves the primitive equations under Boussinesq and hydrostatic approximation using the finite difference numerical schemes. CROCO has higher-order advection schemes which enable better representations of turbulent activities. A third-order upstream-biased horizontal advection scheme, which reduces dispersion errors and enhances the precision for a given grid resolution, has been used^74^. A spline advection scheme, which is equivalent to a conventional scheme of order 8, has been adopted for the vertical momentum advection. For the tracers, a fourth-order Akima scheme has been utilized. The subgrid-scale vertical mixing has been parameterized using the GLS mixing scheme^75^.

The grid setup (Fig. 1) is rotated anticlockwise by 14.5°. This domain receives input from the Agulhas Current at the eastern boundary perpendicularly which leads to less numerical noise. The horizontal resolution of this grid is 3 km, with 50 vertical levels. This spatial resolution is sufficient for studying the mesoscale physical features driving the chlorophyll patterns observed from space at 4 km resolution. The bathymetry is constructed from the hydrographic chart made by the South African Navy Hydrographic Office (SANHO) and then interpolated to the model grid using nearest neighbour interpolation. A local smoothing has further been applied where the topography steepness exceeds a factor, $r = {{\Delta h} \mathord{\left/ {\vphantom {{\Delta h} h}} \right. \kern-\nulldelimiterspace} h}$ by 0.25 (where $h$ is the depth). Figure 1 shows the smoothed topography that is used in the simulation.

The model is initialized and forced at the lateral boundary conditions using the daily HYCOM ocean reanalysis data from the Global Ocean Forecasting System (GOFS) 3.1, obtained from the Naval Research Laboratory: Ocean Dynamics and Prediction Branch. The model is forced at the surface using the 6-hourly Climate Forcing Reanalysis (CFSR) version 1 (available at 0.3° spatial resolution) and version 2 (0.2° spatial resolution) data^76,77^. With this setup, CROCO is then integrated for the period 1 November 2005 to 31 December 2010 using the CFSRv1 forcing and 1 January 2011 to 31 December 2015 using CFSRv2 forcing.

### Model bias correction and assessment

The model temperature and salinity are compared with CTD in situ observations at nine monitoring stations along the St Helena Bay Monitoring Line (SHBML) (Fig. 1) to assess the model performance. These CTD observations are cruise measurements recorded on a quasi-monthly basis performed by the South African Department of Environment, Forestry & Fisheries (DEFF). The supplementary Figs. S5 and S6 show simulated seasonal vertical climatologies of temperature and salinity, respectively and the calculated bias with respect to the climatology obtained from the SHBML observations. This region is typically affected by positive biases in the thermal structure^28^ and salt bias. To lower these errors the monthly-based climatological bias correction was imposed at the boundaries using the anomalies computed between the HYCOM data and the CSIRO Atlas of Regional Seas (CARS 2009) climatology and the model was re-run. The supplementary Figs. S7 and S8 show similar simulated seasonal vertical climatologies and respective bias as presented in supplementary Figs. S5 and S6 after the correction at the boundaries is implemented. The use of the bias-correction method nearly halved the discrepancy from between 2.5 °C–3 °C and 0.24–0.32 psu (Supplementary Figs. S5 and S6) to 1.5–2.5 °C, and 0.16–0.24 psu (Supplementary Figs. S7 and S8). We note that the warm bias is lower near the shelf where super-groups are observed and increases offshore.

A thorough validation of the simulated transport would require an array of current meters or coastal radars which are not available in the region. However, given the baroclinic nature of the Goodhope jet, we can assume that the currents will be dependent on density stratification that can be assessed temporally using the SHBML data. The potential energy anomaly (PEA) has been chosen as the overall proxy for density stratification. The PEA is defined as

2 $$PEA= \frac{1}{H} {\int }_{-H}^{0}\left(\overline{\rho }- \rho \right)gz dz$$

where $\rho$ is the potential density and $\overline{\rho }$ is the vertically averaged potential density^78,79^. PEA represents the amount of energy per unit volume that is required to make the density stratified water column vertically homogeneous. Hence, higher PEA represents strong density stratification. The supplementary Fig. S9 compares the PEA estimated from CROCO simulated salinity and temperature with that estimated from the SHBML observations at station 2 and station 8, respectively. Station 2 is located near the coast, while station 8 is situated ~ 112 km offshore. It is worth mentioning that the observations are available on a monthly basis with some data gaps while simulated fields are saved on a daily basis. For a better comparison of the simulation with the observations, only simulated data of the particular month which is covered by observations are plotted. The PEA estimated from the CROCO simulation is highly correlated with that estimated from the observations at both the stations. The smaller values of PEA near the coast (station 2) are due to the shallow bathymetry. The correlation coefficient between the modelled and observed PEA is 0.43 at station 2 and increases to 0.69 at station 8 at 0.05% significance level (Supplementary Table S1). The lower correlation at station 2 is because of low PEA values and high fluctuations. The correlation between the modelled and observed PEA suggests that CROCO simulates the density stratification properly and the simulated current can be used to estimate the volume transport. To assess the sensitivity to high-frequency and higher resolution atmospheric conditions that may not be captured in the coarse CFSR data, we have compared the PEA time series with a CROCO simulation forced by an hourly atmospheric forcing at 3 km resolution^80^, which is only available until 2012. Supplementary Fig. S10 shows that the results are comparable to Supplementary Fig. S9.

### Estimation of eddy kinetic energy (EKE)

The EKE is estimated from the surface current simulated by CROCO using the following equation:

3 $$EKE=\frac{1}{2}\left[{\left(u- \overline{u }\right)}^{2}+{\left(v- \overline{v }\right)}^{2}\right]$$

where $u$ and $v$ represent zonal and meridional velocity at the surface and $\overline{u }$ and $\overline{v }$ represent the 90 days moving mean of *u* and *v*, respectively. Then the daily EKE is averaged for October to understand the changes in turbulent settings in different years at the focus area.

The significance levels for all the anomalies and confidence intervals for the October EKE were calculated using two-sided *t*-test^81^. In all cases, the sample size is 10, the total number of years.

### Estimation of outward transport and retention

In our results and analysis, we have calculated outward volume transport from the focus region utilizing the CROCO simulation. The transport is calculated in the top 100 m of the water using the following formula.

4 $${T}_{out}= \oint {\int }_{-100}^{0}{v}_{out} dz dl$$

(4) where, $l$ runs through the length of each boundary, and ${v}_{out}$ is the outward velocity from the region which is normal to the respective boundary section at which the transport is estimated.

To compute the retention index the total outward transport through all the boundaries of the focus region is calculated. The negative total outward transport can be considered as a proxy for retention $\left(Ret \sim - {T}_{out}\right)$. The retention index (*RI*) is estimated by using the following normalizing equation of *Ret*.

5 $$RI= \frac{Ret- \overline{Ret}}{{\sigma }_{Ret}}$$

$\overline{Ret }$ and ${\sigma }_{Ret}$ denote the mean and standard deviation, respectively, of average October retention in all the years of model simulation. The outward transport from the region accounts for the nutrients and primary production that is flushed out from the region. The sign changed total outward transport can be considered as the proxy for the retaining nutrient or primary production. The Lilliefors test shows that outward transport is normally distributed at 1% significance level^71^. Moreover, the skewness of the data is 0.04 suggesting the mean of the October outward transport shows little bias towards higher values. This confirms that we may estimate the retention index by normalizing total outward transport.

## Supplementary Information

Supplementary Information.

## Data Availability

The super-groups data are available in Findlay et al. (2017). Ocean colour data are available at https://www.oceancolour.org/. The ASCAT wind stress data are available at https://manati.star.nesdis.noaa.gov/products/ASCAT.php. All other datasets that we presented in this article will be made available upon request. All the model output data are of large size for a public repository and will be made available upon request.

## References

[1] Dawbin WH (1956). The migrations of humpback whales which pass the New Zealand coast. Trans. R. Soc. New Zeal..

[2] Chittleborough R (1965). Dynamics of two populations of the humpback whale, *Megaptera novaeangliae* (Borowski). Mar. Freshw. Res..

[3] Rasmussen K (2007). Southern Hemisphere humpback whales wintering off Central America: Insights from water temperature into the longest mammalian migration. Biol. Let..

[4] Friedlaender AS (2006). Whale distribution in relation to prey abundance and oceanographic processes in shelf waters of the Western Antarctic Peninsula. Mar. Ecol. Prog. Ser..

[5] Nowacek DP (2011). Super-aggregations of krill and humpback whales in Wilhelmina Bay Antarctic Peninsula. PLoS ONE.

[6] Barendse J (2011). Transit station or destination? Attendance patterns, movements and abundance estimate of humpback whales off west South Africa from photographic and genotypic matching. Afr. J. Mar. Sci..

[7] Best PB, Sekiguchi K, Findlay KP (1995). A suspended migration of humpback whales *Megaptera novaeangliae* on the west coast of South Africa. Marine Ecol. Progr. Ser. Oldendorf.

[8] Findlay K, Best P (1995). Summer incidence of humpback whales on the west coast of South Africa. S. Afr. J. Mar. Sci..

[9] Findlay KP (2017). Humpback whale “super-groups”–A novel low-latitude feeding behaviour of Southern Hemisphere humpback whales (*Megaptera novaeangliae*) in the Benguela Upwelling System. PLoS ONE.

[10] Pirotta, V., Owen, K., Donnelly, D., Brasier, M. J. & Harcourt, R. First evidence of bubble‐net feeding and the formation of ‘super‐groups’ by the east Australian population of humpback whales during their southward migration. *Aquat. Conserv.* (2021).

[11] Veitch J, Penven P, Shillington F (2009). The Benguela: A laboratory for comparative modeling studies. Prog. Oceanogr..

[12] Preston-Whyte RA, Tyson PD (1988). Atmosphere and weather of southern Africa.

[13] Nemoto, T., Best, P., Ishimaru, K. & Takano, H. Diatom films on whales [minke whales and 4 species of toothed whales] in South African waters. *Scientific Reports of the Whales Research Institute* (1980).

[14] Hutchings, L., Pitcher, G., Probyn, T. & Bailey, G. in *Upwelling in the ocean: modern processes and ancient records* Vol. 18 (eds CP Summerhayes *et al.*) Ch. 3, 65–81 (Wiley & Sons, 1995).

[15] Clapham, P. J. in *Encyclopedia of marine mammals* (eds B Würsig, JGM Thewissen, & KM Kovacs) 489–492 (Academic Press, 2018).

[16] Bakun A (2015). Anticipated effects of climate change on coastal upwelling ecosystems. Curr. Clim. Change Rep..

[17] Mackas DL, Beaugrand G (2010). Comparisons of zooplankton time series. J. Mar. Syst..

[18] Mackas D (2012). Changing zooplankton seasonality in a changing ocean: Comparing time series of zooplankton phenology. Prog. Oceanogr..

[19] Huggett J, Verheye H, Escribano R, Fairweather T (2009). Copepod biomass, size composition and production in the Southern Benguela: Spatio–temporal patterns of variation, and comparison with other eastern boundary upwelling systems. Prog. Oceanogr..

[20] Verheye HM, Lamont T, Huggett JA, Kreiner A, Hampton I (2016). Plankton productivity of the Benguela current large marine ecosystem (BCLME). Environ. Dev..

[21] Shannon LJ (2020). Exploring temporal variability in the Southern Benguela ecosystem over the past four decades using a time-dynamic ecosystem model. Front. Mar. Sci..

[22] Jarre A (2015). Synthesis: climate effects on biodiversity, abundance and distribution of marine organisms in the Benguela. Fish. Oceanogr..

[23] Lamont T, García-Reyes M, Bograd S, Van Der Lingen C, Sydeman W (2018). Upwelling indices for comparative ecosystem studies: Variability in the Benguela Upwelling System. J. Mar. Syst..

[24] Tim N, Zorita E, Hünicke B (2015). Decadal variability and trends of the Benguela upwelling system as simulated in a high-resolution ocean simulation. Ocean Sci..

[25] Lamont T, Barlow R, Brewin R (2019). Long-term trends in phytoplankton chlorophyll *a* and size structure in the Benguela Upwelling System. J. Geophys. Res. Oceans.

[26] Ragoasha N (2019). Lagrangian pathways in the southern Benguela upwelling system. J. Mar. Syst..

[27] Shannon V, Hempel G, Moloney C, Woods JD, Malanotte-Rizzoli P (2006). Benguela: Predicting a Large Marine Ecosystem.

[28] Veitch J, Penven P, Shillington F (2010). Modeling equilibrium dynamics of the Benguela current system. J. Phys. Oceanogr..

[29] Lachkar Z, Gruber N (2012). A comparative study of biological production in eastern boundary upwelling systems using an artificial neural network. Biogeosciences.

[30] Gruber N (2011). Eddy-induced reduction of biological production in eastern boundary upwelling systems. Nat. Geosci..

[31] Hutchings L (1998). Multiple factors affecting South African anchovy recruitment in the spawning, transport and nursery areas. S. Afr. J. Mar. Sci..

[32] Rossi V, López C, Sudre J, Hernández-García E, Garçon V (2008). Comparative study of mixing and biological activity of the Benguela and Canary upwelling systems. Geophys. Res. Lett..

[33] Barendse J, Best PB (2014). Shore-based observations of seasonality, movements, and group behavior of southern right whales in a nonnursery area on the South African west coast. Mar. Mamm. Sci..

[34] Barendse J (2010). Migration redefined? Seasonality, movements and group composition of humpback whales Megaptera novaeangliae off the west coast of South Africa. Afr. J. Mar. Sci..

[35] Gibbons, M. J. *An introduction to the Zooplankton of the Benguella current Region*. (1997).

[36] Olsen, Ø. *Hvaler og hvalfangst i Sydafrika*. 1–56 (Bergens Museums Arbok 1914–1915, 1914).

[37] Meynecke JO (2020). Responses of humpback whales to a changing climate in the Southern Hemisphere: Priorities for research efforts. Mar. Ecol..

[38] Stockin KA, Burgess EA (2005). Opportunistic Feeding of an Adult Humpback Whale (*Megaptera novaeangliae*) Migrating Along the Coast of Southeastern Queensland, Australia. Aquat. Mamm..

[39] Visser F, Hartman KL, Pierce GJ, Valavanis VD, Huisman J (2011). Timing of migratory baleen whales at the Azores in relation to the North Atlantic spring bloom. Mar. Ecol. Prog. Ser..

[40] Trudelle L (2016). Influence of environmental parameters on movements and habitat utilization of humpback whales (*Megaptera novaeangliae*) in the Madagascar breeding ground. R. Soc. Open Sci..

[41] Veitch J, Hermes J, Lamont T, Penven P, Dufois F (2018). Shelf-edge jet currents in the southern Benguela: A modelling approach. J. Mar. Syst..

[42] Hutchings L (2009). The Benguela current: An ecosystem of four components. Prog. Oceanogr..

[43] Rockwood RC, Elliott ML, Saenz B, Nur N, Jahncke J (2020). Modeling predator and prey hotspots: Management implications of baleen whale co-occurrence with krill in Central California. PLoS ONE.

[44] Hayward TL, Venrick EL (1998). Nearsurface pattern in the California Current: Coupling between physical and biological structure. Deep Sea Res. Part II.

[45] Croll DA (2005). From wind to whales: trophic links in a coastal upwelling system. Mar. Ecol. Prog. Ser..

[46] Walker D, Peterson W (1991). Relationships between hydrography, phytoplankton production, biomass, cell size and species composition, and copepod production in the southern Benguela upwelling system in April 1988. S. Afr. J. Mar. Sci..

[47] Stuart V, Pillar S (1990). Diel grazing patterns of all ontogenetic stages of Euphausia lucens and in situ predation rates on copepods in the southern Benguela upwelling region. Mar. Ecol. Progr. Ser..

[48] Clapham, P. & Baker, C. (Academic, New York, 2002).

[49] Shannon LJ, Field JG, Moloney CL (2004). Simulating anchovy–sardine regime shifts in the southern Benguela ecosystem. Ecol. Model..

[50] Lett C, Roy C, Levasseur A, Van Der Lingen CD, Mullon C (2006). Simulation and quantification of enrichment and retention processes in the southern Benguela upwelling ecosystem. Fish. Oceanogr..

[51] Branch TA (2011). Humpback whale abundance south of 60°S from three complete circumpolar sets of surveys. J. Cetacean Res. Manage..

[52] Findlay K, Best P, Meÿer M (2011). Migrations of humpback whales past Cape Vidal, South Africa, and an estimate of the population increase rate (1988–2002). Afr. J. Mar. Sci..

[53] Henson SA, Cole HS, Hopkins J, Martin AP, Yool A (2018). Detection of climate change-driven trends in phytoplankton phenology. Glob. Change Biol..

[54] Carvalho I (2014). Does temporal and spatial segregation explain the complex population structure of humpback whales on the coast of West Africa?. Mar. Biol..

[55] Kershaw F (2017). Multiple processes drive genetic structure of humpback whale (Megaptera novaeangliae) populations across spatial scales. Mol. Ecol..

[56] Korrûbel J (1992). An age-structured simulation model to investigate species replacement between pilchard and anchovy populations in the southern Benguela. S. Afr. J. Mar. Sci..

[57] Shannon L (1992). The 1980s–a decade of change in the Benguela ecosystem. S. Afr. J. Mar. Sci..

[58] Verheye H, Richardson A, Hutchings L, Marska G, Gianakouras D (1998). Long-term trends in the abundance and community structure of coastal zooplankton in the southern Benguela system, 1951–1996. Afr. J. Mar. Sci..

[59] Bakun A (1990). Global climate change and intensification of coastal ocean upwelling. Science.

[60] Sydeman W (2014). Climate change and wind intensification in coastal upwelling ecosystems. Science.

[61] Bonino G, Di Lorenzo E, Masina S, Iovino D (2019). Interannual to decadal variability within and across the major eastern boundary upwelling systems. Sci. Rep..

[62] Fearon G (2020). Enhanced vertical mixing in coastal upwelling systems driven by diurnal-inertial resonance: Numerical experiments. J. Geophys. Res. Oceans.

[63] Xiu P, Chai F, Curchitser EN, Castruccio FS (2018). Future changes in coastal upwelling ecosystems with global warming: The case of the California Current System. Sci. Rep..

[64] Roxy MK (2016). A reduction in marine primary productivity driven by rapid warming over the tropical Indian Ocean. Geophys. Res. Lett..

[65] Lockerbie EM, Shannon L (2019). Toward exploring possible future states of the southern Benguela. Front. Mar. Sci..

[66] Ortega-Cisneros K, Cochrane KL, Fulton EA, Gorton R, Popova E (2018). Evaluating the effects of climate change in the southern Benguela upwelling system using the Atlantis modelling framework. Fish. Oceanogr..

[67] Rykaczewski RR, Checkley DM (2008). Influence of ocean winds on the pelagic ecosystem in upwelling regions. Proc. Natl. Acad. Sci..

[68] Veitch JA, Penven P (2017). The role of the A gulhas in the B enguela current system: A numerical modeling approach. J. Geophys. Res. Oceans.

[69] Beal LM, De Ruijter WP, Biastoch A, Zahn R (2011). On the role of the Agulhas system in ocean circulation and climate. Nature.

[70] Beal LM, Elipot S (2016). Broadening not strengthening of the Agulhas current since the early 1990s. Nature.

[71] Lilliefors HW (1969). On the Kolmogorov-Smirnov test for the exponential distribution with mean unknown. J. Am. Stat. Assoc..

[72] Shchepetkin AF, McWilliams JC (2005). The regional oceanic modeling system (ROMS): A split-explicit, free-surface, topography-following-coordinate oceanic model. Ocean Model.

[73] Debreu L, Marchesiello P, Penven P, Cambon G (2012). Two-way nesting in split-explicit ocean models: Algorithms, implementation and validation. Ocean Model.

[74] Shchepetkin AF, McWilliams JC (1998). Quasi-monotone advection schemes based on explicit locally adaptive dissipation. Mon. Weather Rev..

[75] Warner JC, Sherwood CR, Arango HG, Signell RP (2005). Performance of four turbulence closure models implemented using a generic length scale method. Ocean Model.

[76] Saha S (2010). NCEP Climate Forecast System Reanalysis (CFSR) 6-Hourly Products, January 1979 to December 2010.

[77] Saha S (2011). NCEP Climate Forecast System Version 2 (CFSv2) 6-hourly Products. D61C61TXF.

[78] Burchard H, Hofmeister R (2008). A dynamic equation for the potential energy anomaly for analysing mixing and stratification in estuaries and coastal seas. Estuar. Coast. Shelf Sci..

[79] Yamaguchi R, Suga T, Richards KJ, Qiu B (2019). Diagnosing the development of seasonal stratification using the potential energy anomaly in the North Pacific. Clim. Dyn..

[80] Lennard C, Hahmann AN, Badger J, Mortensen NG, Argent B (2015). Development of a numerical wind atlas for South Africa. Energy Proc..

[81] Thomson RE, Emery WJ (2014). Data Analysis Methods in Physical Oceanography.

