# Supplementary material for: Oceanographic anomalies coinciding with humpback whale super-group occurrences in the Southern Benguela
Source: Sci Rep. 2021 Oct 22;11:20896. doi: 10.1038/s41598-021-00253-2 (PMC8536746; doi:10.1038/s41598-021-00253-2)
Supplement: Supplementary file 1 — Supplementary Information. [file 41598_2021_253_MOESM1_ESM.pdf]

Supplementary figures for “Oceanographic anomalies coinciding with humpback whale super-group occurrences in the Southern Benguela”

Subhra Prakash Dey[1,2,*], Marcello Vichi[1,2,*], Giles Fearon[1,2], Elisa Seyboth[3], Ken P. Findlay[3], Jan-Olaf Meynecke[4,5], Jasper de Bie[4,5], Serena Blyth Lee[4,5], Saumik Samanta[6], Jan-Lukas Menzel Barraqueta[6], Alakendra N. Roychoudhury[6], Brendan Mackey[4]

Affiliations
1. Department of Oceanography, University of Cape Town, 7701 Rondebosch, South Africa
2. Marine and Antarctic Research centre for Innovation and Sustainability, University of Cape Town, 7701 Rondebosch, South Africa
3. Centre for Sustainable Oceans, Faculty of Applied Sciences, Cape Peninsula University of Technology, Cape Town, South Africa
4. Griffith Climate Change Response Program, Griffith University, Southport, Qld, Australia
5. Coastal and Marine Research Centre, Griffith University, Southport, Qld, Australia
6. Earth Sciences, Stellenbosch University, Cape Town, South Africa

[*]Corresponding Authors

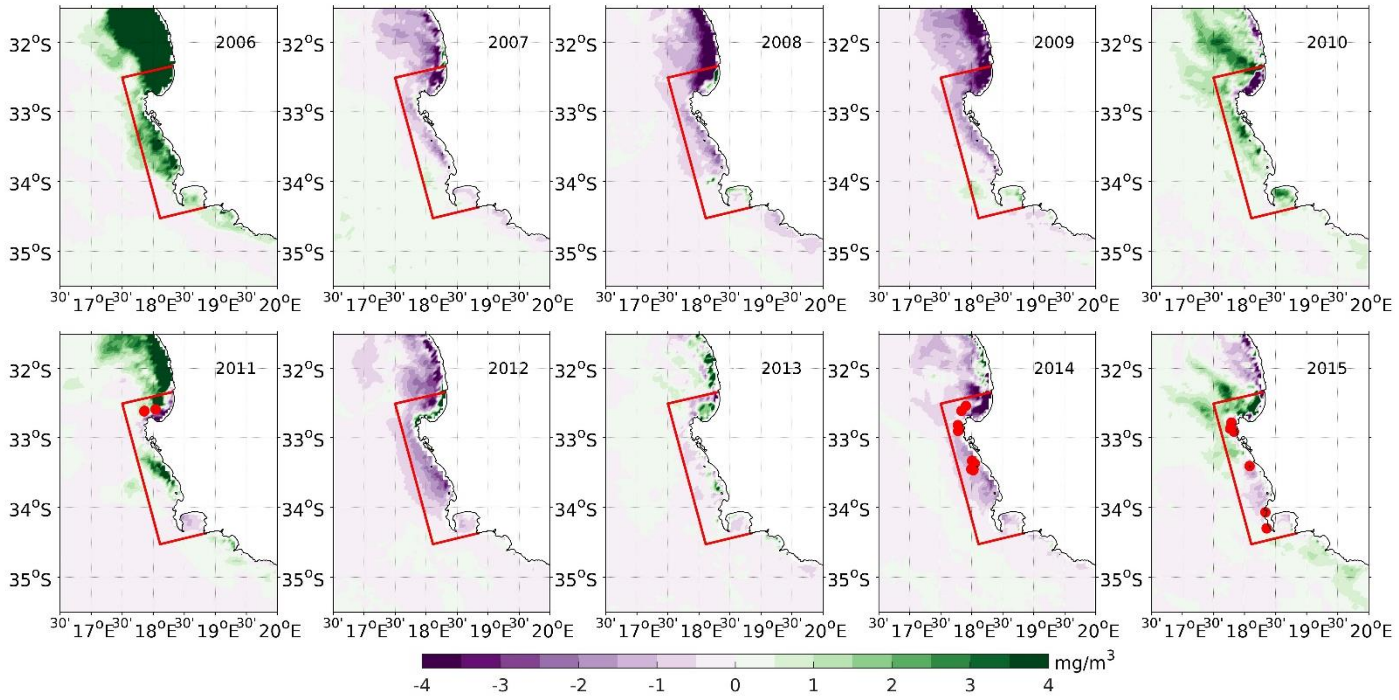

Supplementary figure S1. Chlorophyll-a monthly anomaly (*mg/m³*) in September in years 2006 – 2015. The red dots represent the locations of the super-groups in 2011, 2014 and 2015. The area enclosed by the red lines represents the focus area. The plots are generated using MATLAB 2020b (https://matlab.mathworks.com/) with M_Map, a mapping package available at https://www.eoas.ubc.ca/~rich/map.html.

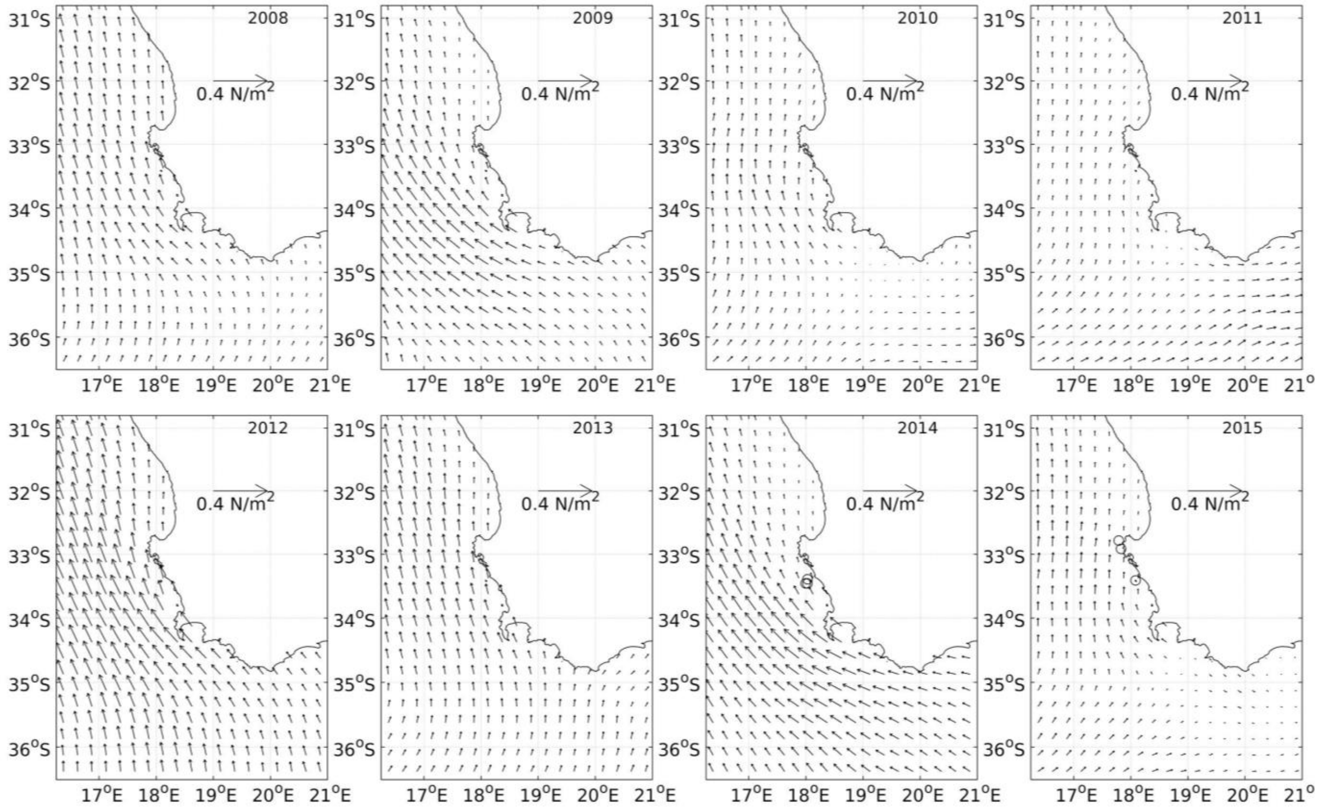

Supplementary figure S2. Wind stress in October in different years averaged from the wind fields measured by ASCAT. Weak wind stress in 2010, 2011, 2014 and 2015. Super-group years: 2011, 2014, and 2015. The plots are generated using MATLAB 2020b (https://matlab.mathworks.com/) with M_Map, a mapping package available at https://www.eoas.ubc.ca/~rich/map.html.

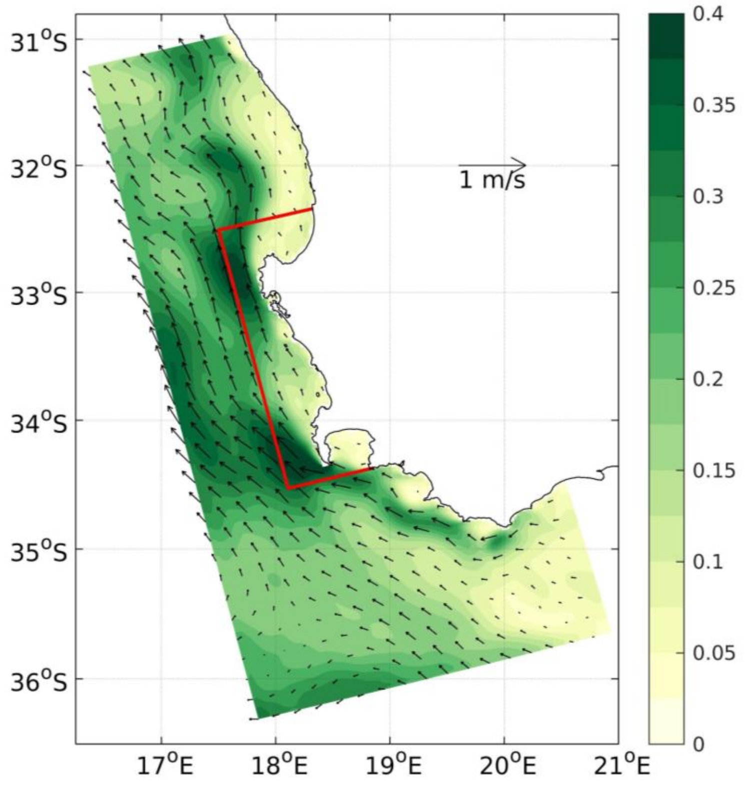

Supplementary figure S3. Climatological surface ocean current (vector) and speed (color, *m/s*) in October. The region enclosed by the red lines represents the focus area. This plot is generated using MATLAB 2020b (https://matlab.mathworks.com/) with M_Map, a mapping package available at https://www.eoas.ubc.ca/~rich/map.html.

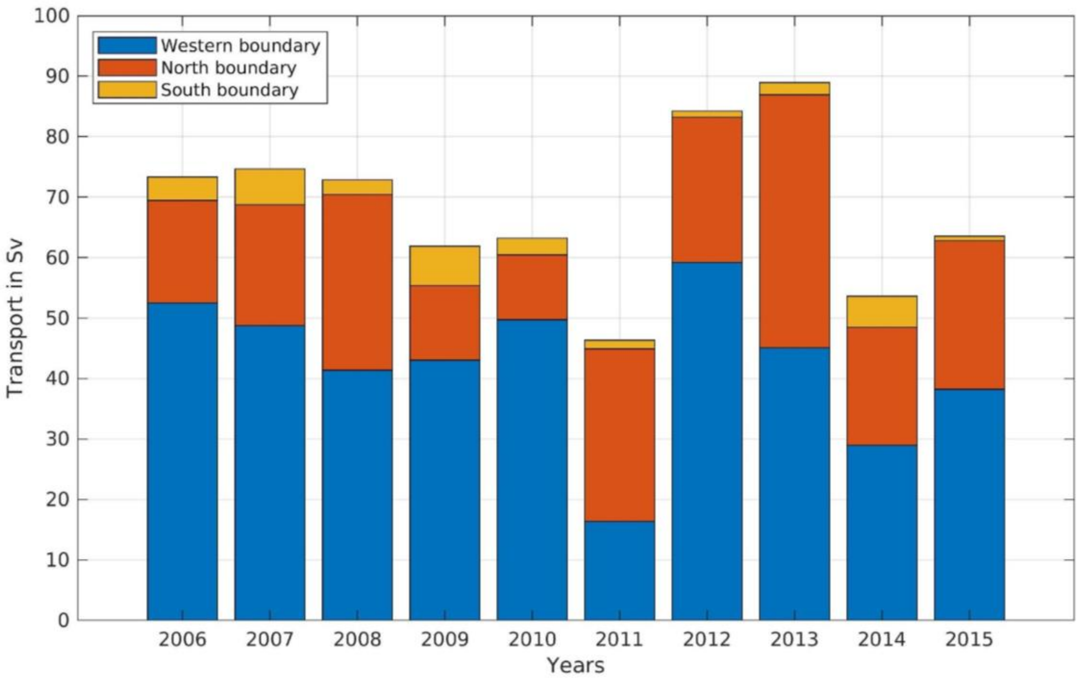

Supplementary figure S4. The outward volume transport in Sv in October from the analysis region through the western (blue bars), northern (red bars) and southern (orange bars) boundaries. Note reduced outward transport in super-group years: 2011, 2014, and 2015.

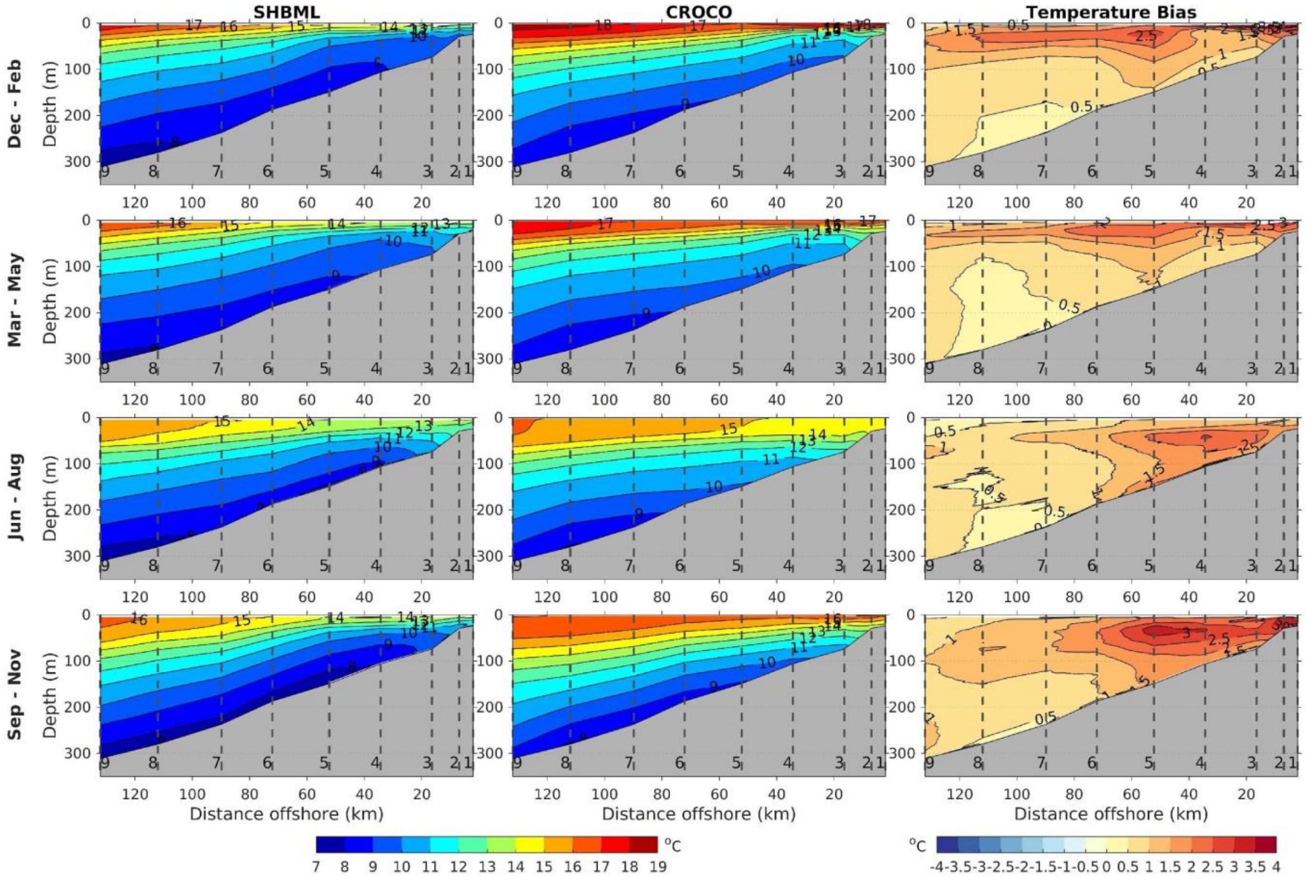

Supplementary figure S5. Vertical section of seasonal climatological temperature estimated from (a) SHBML observations, (b) CROCO simulation along the St. Helena Bay monitoring line without boundary correction. (c) Vertical section of temperature bias (CROCO – SHBML obs) along the same monitoring line.

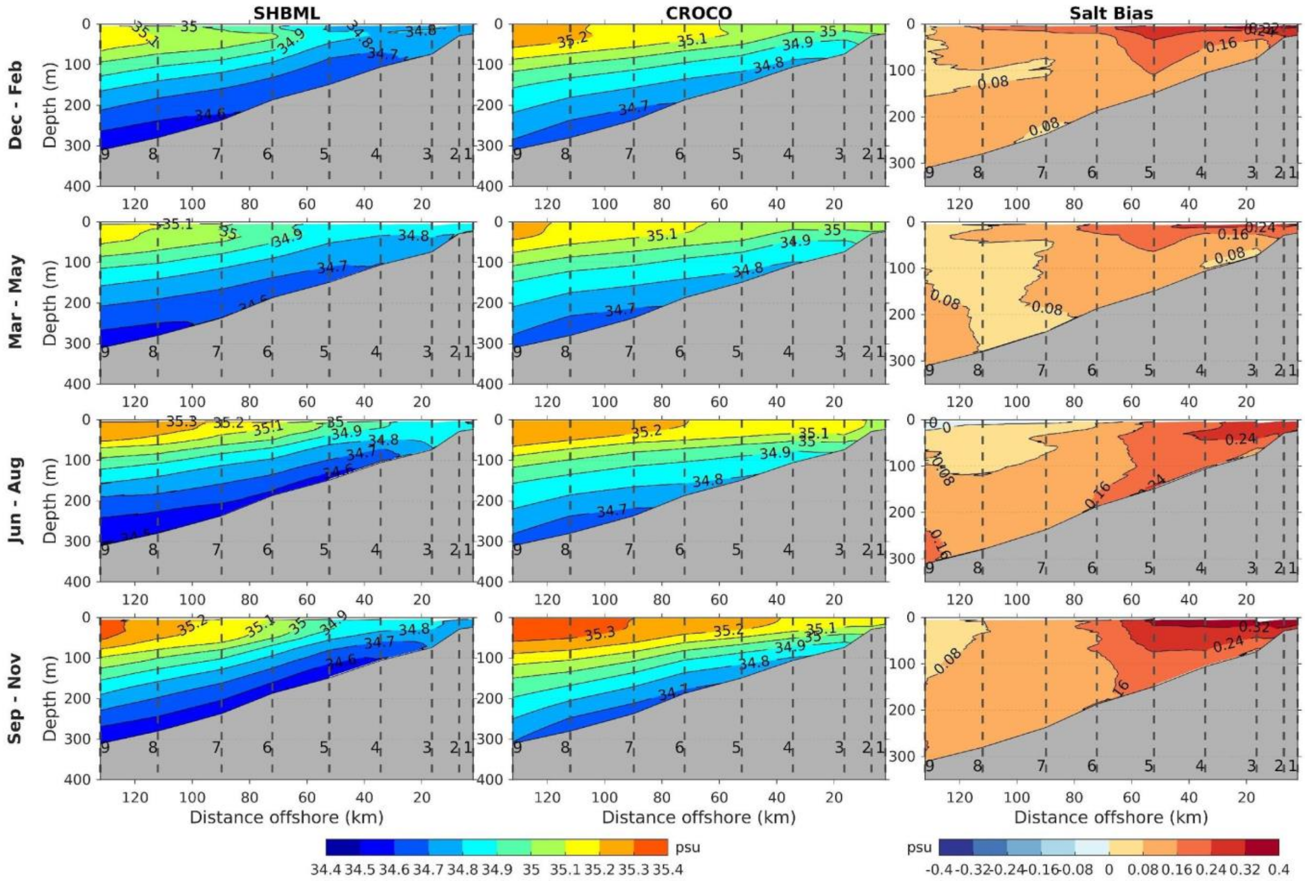

Supplementary figure S6. Vertical section of seasonal climatological salinity estimated from (a) SHBML observations, (b) CROCO simulation without boundary correction along the St. Helena Bay monitoring line. (c) Vertical section of salinity bias (CROCO – SHBML obs) along the same monitoring line.

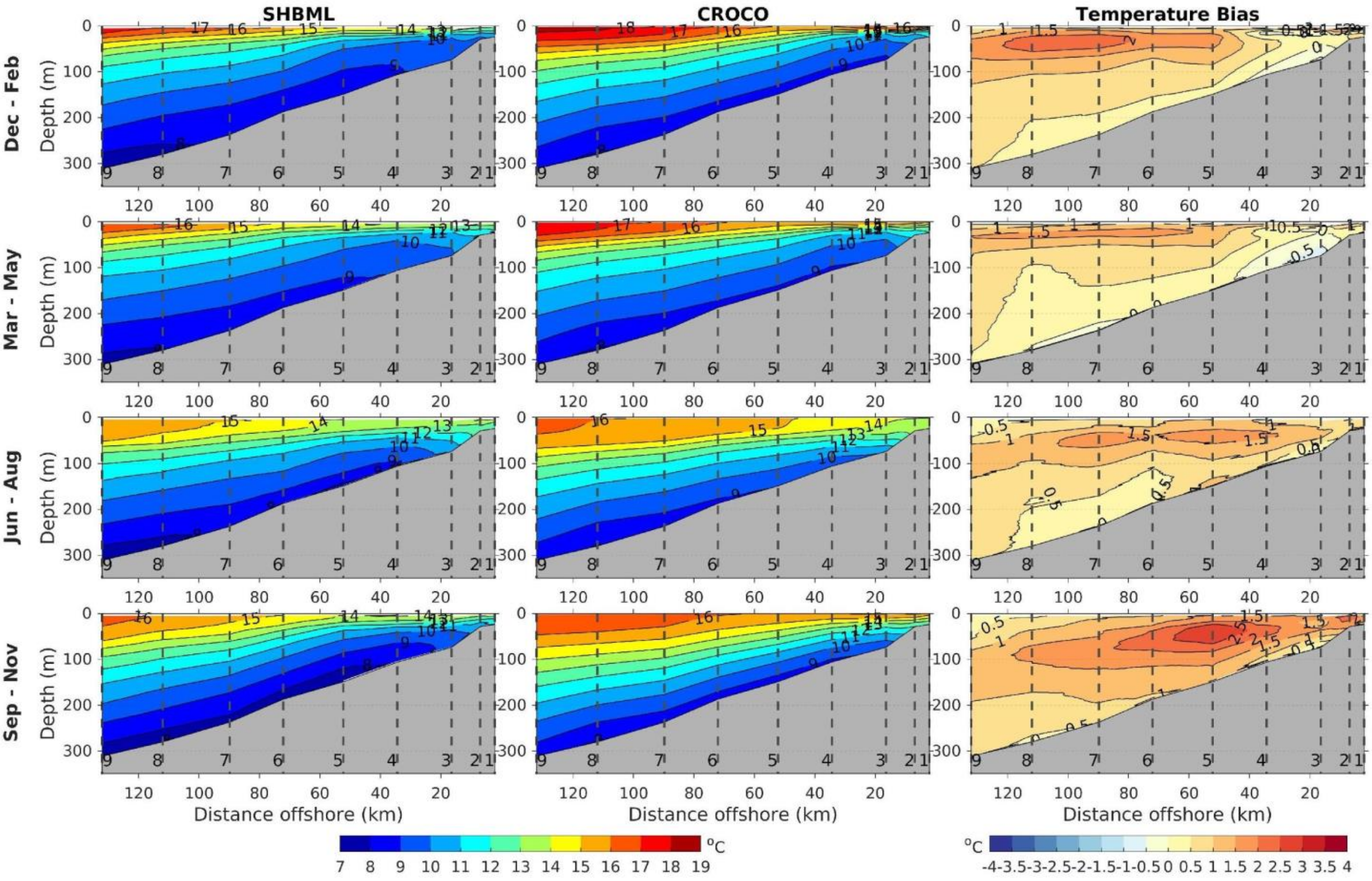

Supplementary figure S7. Vertical section of seasonal climatological temperature estimated from (a) SHBML observations, (b) CROCO simulation with boundary correction along the St. Helena Bay monitoring line. (c) Vertical section of temperature bias (CROCO – SHBML observations) along the same monitoring line.

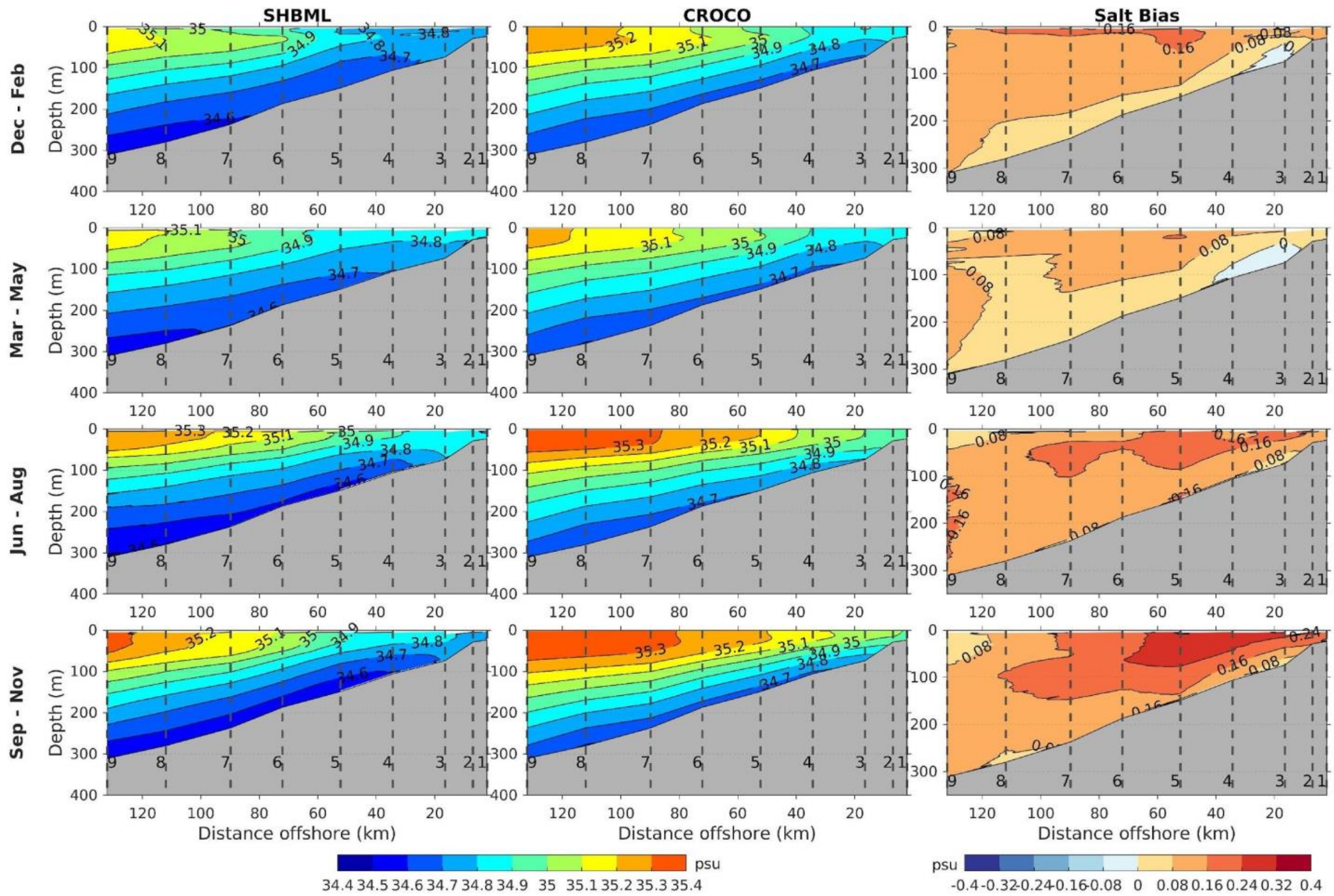

Supplementary figure S8. Vertical section of seasonal climatological salinity estimated from (a) SHBML observations, (b) CROCO simulation with boundary correction along the St. Helena Bay monitoring line. (c) Vertical section of salinity bias (CROCO – SHBML obs) along the same monitoring line.

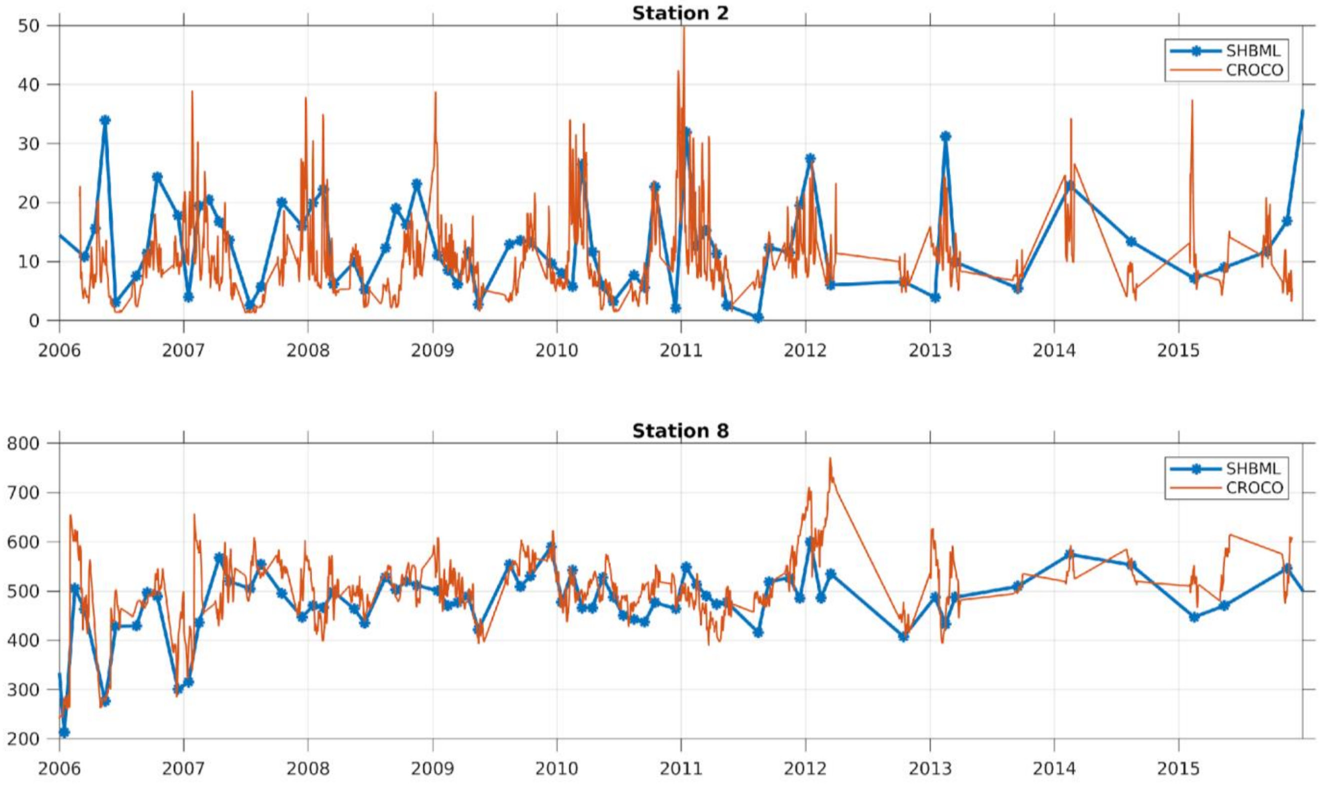

Supplementary figure S9. Variation of PEA estimated from the SHBML observations (blue line), and CROCO simulation (red line) in two stations: (a) station 2, situated near the shoreline, and (b) station 8, situated near 112 km offshore in the open ocean.

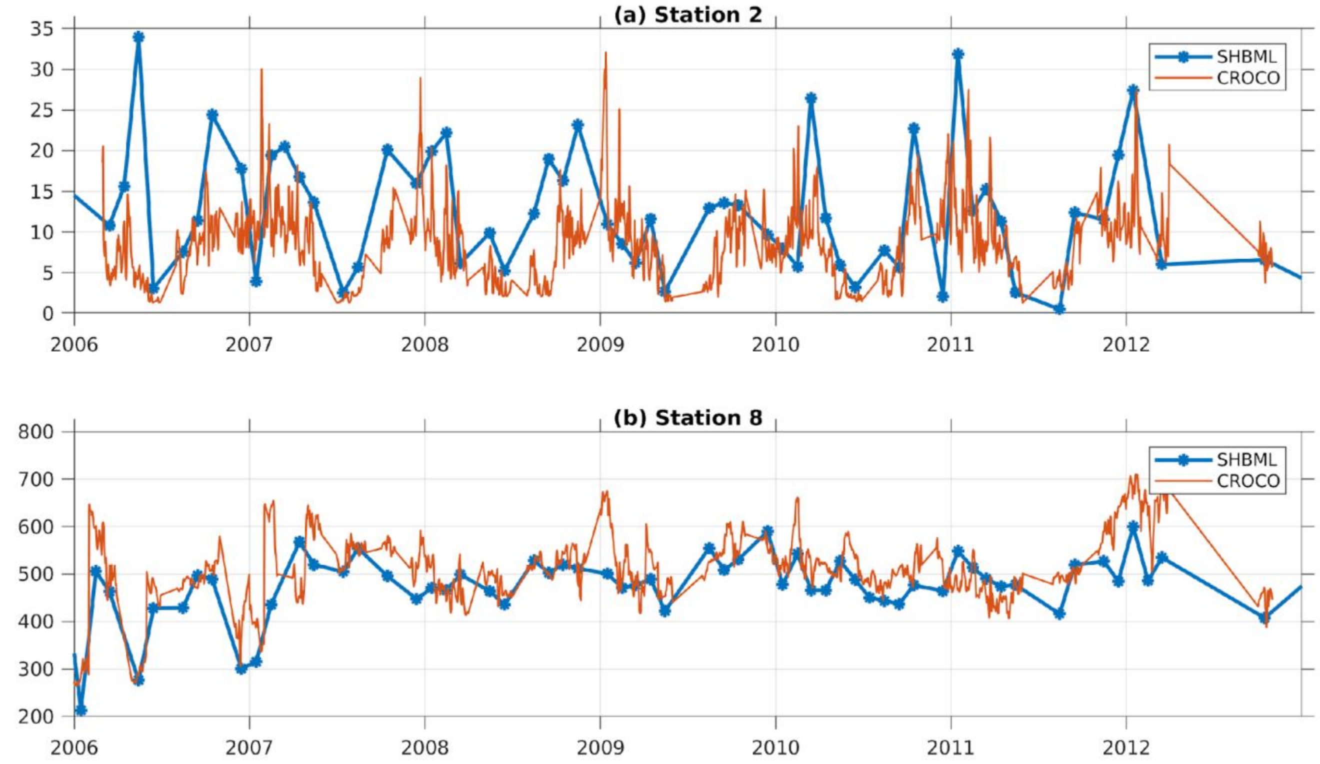

Supplementary figure S10. Comparison of PEA variation estimated from the SHBML observations (blue line), and CROCO simulation (red line) forced by high-resolution CSAG atmospheric forcing in two stations: (a) station 2, situated near the shoreline, and (b) station 8, situated near 112 km offshore in the open ocean.

Supplementary Table S1: Statistical metrics to assess the CROCO performance in estimating PEA at station 2 and station 8.

Station	CROCO Mean	SHBML Mean	Correlation	$RMSD_{TOT}$	$RMSD_{CP}$	Bias	AAE
Station 1	9.93	12.77	0.43	7.98	7.46	-2.84	6.29
Station 8	507.51	479.62	0.69	63.75	57.32	27.89	48.35
